# Risk of Subsequent Stroke Among Patients Receiving Outpatient vs Inpatient Care for Transient Ischemic Attack: A Systematic Review and Meta-analysis

**DOI:** 10.1001/jamanetworkopen.2021.36644

**Published:** 2022-01-05

**Authors:** Shima Shahjouei, Jiang Li, Eric Koza, Vida Abedi, Alireza Vafaei Sadr, Qiushi Chen, Ashkan Mowla, Paul Griffin, Annemarei Ranta, Ramin Zand

**Affiliations:** 1 Neurology Department, Neuroscience Institute, Geisinger Health System, Danville, Pennsylvania; 2 Department of Molecular and Functional Genomics, Geisinger Health System, Danville, Pennsylvania; 3 Geisinger Commonwealth School of Medicine, Scranton, Pennsylvania; 4 Biocomplexity Institute, Virginia Tech, Blacksburg, Virginia; 5 Department de Physique Theorique and Center for Astroparticle Physics, University Geneva, Geneva, Switzerland; 6 Department of Industrial and Manufacturing Engineering, Pennsylvania State University, University Park; 7 Division of Stroke and Endovascular Neurosurgery, Department of Neurological Surgery, Keck School of Medicine, University of Southern California, Los Angeles; 8 Department of Neurology, Wellington Hospital, Wellington, New Zealand; 9 Department of Medicine, University of Otago, Wellington, New Zealand

## Abstract

**Importance:**

Transient ischemic attack (TIA) often indicates a high risk of subsequent cerebral ischemic events. Timely preventive measures improve the outcome.

**Objective:**

To estimate and compare the risk of subsequent ischemic stroke among patients with TIA or minor ischemic stroke (mIS) by care setting.

**Data Sources:**

MEDLINE, Web of Science, Scopus, Embase, International Clinical Trials Registry Platform, ClinicalTrials.gov, Trip Medical Database, CINAHL, and all Evidence-Based Medicine review series were searched from the inception of each database until October 1, 2020.

**Study Selection:**

Studies evaluating the occurrence of ischemic stroke after TIA or mIS were included. Cohorts without data on evaluation time for reporting subsequent stroke, with retrospective diagnosis of the index event after stroke occurrence, and with a report of outcomes that were not limited to patients with TIA or mIS were excluded. Two authors independently screened the titles and abstracts and provided the list of candidate studies for full-text review; discrepancies and disagreements in all steps of the review were addressed by input from a third reviewer.

**Data Extraction and Synthesis:**

The study was prepared and reported following the Preferred Reporting Items for Systematic Reviews and Meta-analyses, Meta-analysis of Observational Studies in Epidemiology, Methodological Expectations of Cochrane Intervention Reviews, and Enhancing the Quality and Transparency of Health Research guidelines. The Risk of Bias in Nonrandomized Studies—of Exposures (ROBINS-E) tool was used for critical appraisal of cohorts, and funnel plots, Begg-Mazumdar rank correlation, Kendall τ^2^, and the Egger bias test were used for evaluating the publication bias. All meta-analyses were conducted under random-effects models.

**Main Outcomes and Measures:**

Risk of subsequent ischemic stroke among patients with TIA or mIS who received care at rapid-access TIA or neurology clinics, inpatient units, emergency departments (EDs), and unspecified or multiple settings within 4 evaluation intervals (ie, 2, 7, 30, and 90 days).

**Results:**

The analysis included 226 683 patients from 71 articles recruited between 1981 and 2018; 5636 patients received care at TIA clinics (mean [SD] age, 65.7 [3.9] years; 2291 of 4513 [50.8%] men), 130 139 as inpatients (mean [SD] age, 78.3 [4.0] years; 49 458 of 128 745 [38.4%] men), 3605 at EDs (mean [SD] age, 68.9 [3.9] years; 1596 of 3046 [52.4%] men), and 87 303 patients received care in an unspecified setting (mean [SD] age, 70.8 [3.8] years, 43 495 of 87 303 [49.8%] men). Among the patients who were treated at a TIA clinic, the risk of subsequent stroke following a TIA or mIS was 0.3% (95% CI, 0.0%-1.2%) within 2 days, 1.0% (95% CI, 0.3%-2.0%) within 7 days, 1.3% (95% CI, 0.4%-2.6%) within 30 days, and 2.1% (95% CI, 1.4%-2.8%) within 90 days. Among the patients who were treated as inpatients, the risk of subsequent stroke was to 0.5% (95% CI, 0.1%-1.1%) within 2 days, 1.2% (95% CI, 0.4%-2.2%) within 7 days, 1.6% (95% CI, 0.6%-3.1%) within 30 days, and 2.8% (95% CI, 2.1%-3.5%) within 90 days. The risk of stroke among patients treated at TIA clinics was not significantly different from those hospitalized. Compared with the inpatient cohort, TIA clinic patients were younger and had had lower ABCD^2^ (age, blood pressure, clinical features, duration of TIA, diabetes) scores (inpatients with ABCD^2^ score >3, 1101 of 1806 [61.0%]; TIA clinic patients with ABCD^2^ score >3, 1933 of 3703 [52.2%]).

**Conclusions and Relevance:**

In this systematic review and meta-analysis, the risk of subsequent stroke among patients who were evaluated in a TIA clinic was not higher than those hospitalized. Patients who received treatment in EDs without further follow-up had a higher risk of subsequent stroke. These findings suggest that TIA clinics can be an effective component of the TIA care component pathway.

## Key Points

**Question:**

Does the risk of subsequent stroke differ by care setting among patients with transient ischemic attack (TIA) or minor stroke?

**Findings:**

In this systematic review and meta-analysis of 226 683 unique patients in 71 unique studies, patients cared for in a TIA clinic vs as inpatients had similar risks of subsequent stroke. Patients who were treated in emergency departments without further follow-up had a higher risk of subsequent stroke than those treated as inpatients or in TIA clinics.

**Meaning:**

In this study, the risk of subsequent stroke among patients who received treatment in a TIA clinic was not higher than those who were hospitalized.

## Teaser

This systematic review and meta-analysis compares the risk of subsequent ischemic stroke among patients with transient ischemic attack (TIA) or minor ischemic stroke who received care at rapid-access TIA or neurology clinics, inpatient units, emergency departments, and unspecified or multiple settings.

## Introduction

Studies have shown up to an 80% reduction in the risk of stroke after a transient ischemic attack (TIA) with early implementation of secondary stroke prevention strategies.^1,2,3^ Our study^4^ examining the trends in TIA outcome during the past 5 decades indicated that the risk of subsequent stroke has remained unchanged since 1999.

Despite the need for an urgent investigation of the etiology and initiation of preventive measures for patients with TIA, there is no consensus on the care pathway protocol. The evaluation and hospitalization rates after TIA vary widely among practitioners, hospitals, and regions.^5,6,7,8^ Several TIA care pathway models have been proposed mainly to reduce the hospital length of stay and admission costs and to improve outcomes.^9,10,11^ Several studies have indicated that the outpatient management of TIA among selected patients can be safe and cost-effective.^1,9,10,12,13,14,15^ Nevertheless, in many instances outpatient care for selected patients with TIA is avoided.

There is no comprehensive study comparing the outcome of patients with TIA who received care in different settings. The goal of the current meta-analysis was to estimate and compare the risk of subsequent ischemic stroke among patients with TIA or minor ischemic stroke (mIS) who received care at rapid access TIA or neurology clinics, inpatient units, emergency departments (EDs), and unspecified or multiple settings within 4 evaluation intervals (2, 7, 30, and 90 days).

## Methods

We prepared and reported the present study according to Preferred Reporting Items for Systematic Reviews and Meta-Analyses (PRISMA),^16^ Meta-analysis of Observational Studies in Epidemiology (MOOSE),^17^ Methodological Expectations of Cochrane Intervention Reviews (MECIR),^18^ and Enhancing the Quality and Transparency of Health Research (EQUATOR)^19^ guidelines.

### Search Strategy

We identified potentially eligible studies by systematically searching the databases Medline, Web of Science, Scopus, Embase, International Clinical Trials Registry Platform (ICTRP), ClinicalTrials.gov, Trip Medical Database, CINAHL, and all Evidence-Based Medicine review series (Cochrane Database of Systematic Reviews, ACP Journal Club, Database of Abstracts of Reviews of Effects, Cochrane Clinical Answers, Cochrane Central Register of Controlled Trials, Cochrane Methodology Register, Health Technology Assessment, and NHS Economic Evaluation Database) (eAppendix in the Supplement). The search queries were primarily conducted from the inception of each database until October 1, 2020, without restriction on study design, document type, language, or socioeconomic and health-expenditure indices of the publishing institute. To minimize the risk of publication bias, peer-reviewed publications, unpublished studies, and gray literature sources were evaluated. We augmented the search results by manually forward and backward citation tracking (in Google Scholar) and communication with selected authors.

### Eligibility Criteria

All studies providing information on the occurrence of ischemic stroke after TIA or mIS (index event) were recorded. We included retrospective and prospective cohorts of adult patients, with both the time-based^20^ and the tissue-based^21^ definitions of TIA as well as alternative definitions of mIS, as National Institutes of Health Stroke Scale (NIHSS) score of 3 or less,^22^ persistence of symptoms for at least 24 hours, or positive diffusion-weighted imaging within 24 hours of symptom onset.^21^ We excluded cohorts (1) without available evaluation time for reporting subsequent stroke, (2) with retrospective diagnosis of index event after stroke occurrence, (3) with a report of outcomes for all triaged patients not limited to TIA or mIS, and (4) duplicate reports.

### Outcome Measure

The outcome of the study was the proportion of early ischemic strokes after the index TIA or mIS among patients who received acute care management in 4 settings: (1) TIA clinic, defined as rapid-access TIA or neurology clinics in which a patient was evaluated within 2 weeks of symptom onset; (2) inpatient, defined as medical-surgical units, stroke units, or observation units; (3) ED, defined as cohorts of patients receiving care in an ED without referral to the TIA clinic or hospitalization; and (4) unspecified setting, including combined reports of outcome from different settings when they could not be differentiated and multicenter studies without a unique protocol. We considered the comparison between the outcomes of patients treated in a TIA clinic vs as inpatients as our main interest. Admissions to in-hospital observation units (ie, <24 hours), although often seen as an outpatient visit (for billing purposes), were considered inpatient due to similarities in the protocols. We reported the outcomes of each setting within 2, 7, 30, and 90 days.

### Screening and Data Extraction

Two reviewers (S.S. and E.K.) independently screened the titles and abstracts and provided the list of candidate studies for full-text review. We addressed the discrepancies and disagreements in all steps of the review by input from a third reviewer (R.Z.). The output of the search was compiled in Mendeley version 1.19.6. Duplicate sets were removed. Records in languages other than English were screened by native speakers. For each study, the data regarding each cohort of patients who received acute care in a similar setting were recorded separately.

### Risk-of-Bias and Publication Bias Assessment

We applied the Risk of Bias in Nonrandomized Studies—of Exposures (ROBINS-E) tool^23,24^ for critical appraisal of the cohorts. The assessment was recorded as low, moderate, or high risk of bias or no information. The degree of bias was measured by the Begg-Mazumdar rank correlation Kendall τ^2^ and the Egger bias test.^25^

### Statistical Analysis

To explore the differences among the estimators, we used (1) moment estimators, ie, DerSimonian and Laird (DL), Hunter and Schmidt (HS), and Hedges (HE); (2) maximum likelihood estimators, ie, maximum likelihood (ML) and restricted maximum likelihood (REML); (3) model error variance estimator, ie, Sidik and Jonkman (SJ); and (4) Bayes estimator, ie, empirical Bayes (EB).^25^

We explored the possible moderator effect of (1) acute-care setting, (2) evaluation intervals, (3) study design of each cohort, (4) recruitment interval (ie, before 2000, 2000-2007, and after 2007, based on the pioneer guidelines in TIA care^1,13,26,27^), and (5) age, blood pressure, clinical features, duration of TIA, diabetes (ABCD^2^) score (percentage of patients in each cohort with ABCD^2^ score of <4 vs ≥4) on the outcome (risk of subsequent stroke) through mixed-effect models using REML as an estimator.^28^ Omnibus test was used to compare the models vs null hypothesis. We compared the outcome of each moderator by calculating the risk of subsequent stroke, between-group *I*^2^, residual heterogeneity, and *P* value (eTable 1 in the Supplement). We assessed the subsequent stroke risk estimates for each evaluation interval separately and considered the setting of care as a subclass under each evaluation interval. We reevaluated the association of ABCD^2^ score with the outcome under each setting-of-care strata (eTable 2 in the Supplement). We performed sensitivity analysis for evaluating the impact of recruitment interval and study design.

We considered a 2-tailed *P* < .05 as statistically significant in all tests. The difference among subgroups was evaluated by pairwise comparisons and adjusted α level, when applicable. Meta-analyses were performed using R version 4.0.2, metafor package (R Project for Statistical Computing).^28^ Forest plots were reproduced in Python version 3.8 for further validation and better visualization.

## Results

### Literature Review and Study Selection

**Figure 1.**
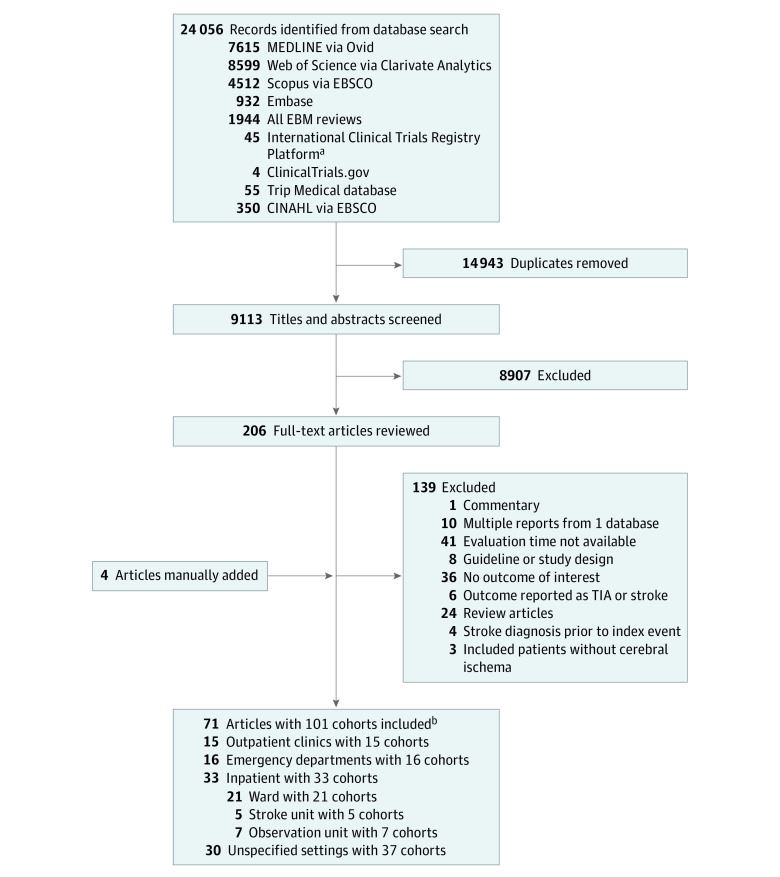
Study Flowchart TIA indicates transient ischemic attack. ^a^EBM reviews include Cochrane Database of Systematic Reviews, ACP Journal Club, Database Abstracts of Reviews and Effects, Cochrane Clinical Answers; Cochrane Central Register of Controlled Trials, Cochrane Methodological Register, Health Technology, and NHS Economic Evaluation Database. ^b^Articles could include multiple settings.

The search protocol resulted in 24 056 records (Figure 1). After the removal of 14 943 duplicate records, the titles and abstracts of 9113 discrete search results were screened. Of the 206 potentially eligible studies, 139 articles were excluded after full-text review (eTable 3 in the Supplement).

Review of the reference lists, citation tracking, and communication with authors led to inclusion of four additional studies. A total of 71 studies were included (A. Mowla, MD, unpublished data, 2020).^9,15,27,29,30,31,32,33,34,35,36,37,38,39,40,41,42,43,44,45,46,47,48,49,50,51,52,53,54,55,56,57,58,59,60,61,62,63,64,65,66,67,68,69,70,71,72,73,74,75,76,77,78,79,80,81,82,83,84,85,86,87,88,89,90,91,92,93,94,95,96^

### Patient Characteristics

This review includes 226 683 patients recruited between 1981 and 2018. Patients were studied prospectively in 24 cohorts (23.8%).^29,30,31,32,33,34,35,36,37,38,39,40,41,42,43,44,45,46,47,48,49^ By considering the health care setting for the index event, we recorded 101 distinct cohorts. Out of 101 cohorts, 16 (15.8%) included patients with TIA and mIS.^27,29,30,32,50,51,52,53,97^ TIA was defined based on a tissue-based definition in 7 studies (9.8%)^33,52,54,55,56,57^ (and A. Mowla, MD, unpublished data, 2020). The Table includes the summary of baseline characteristics and vascular risk factors.

**Table.** Baseline Characteristics and Vascular Risk Factors Among Patients Receiving Care at Each Setting

Characteristic	Patients by acute-care setting, No./total No. available (%)			
	TIA clinic (n = 5636)^a^	Inpatient (n = 130 136)	Emergency department (n = 3605)	Unspecified (n = 87 303)
Age, mean (SD), y	65.7 (3.9)	78.3 (4.0)	68.9 (3.9)	70.8 (3.8)
Men	2291/4513 (50.8)	49 458/128 745 (38.4)	1596/3046 (52.4)^b^	43 495/87 303 (49.8)
Women	2222/4513 (49.2)	79 287/128 745 (61.6)	1450/3046 (47.6)	43 808/87 303 (50.2)
ABCD^2^ score >3	1933/3703 (52.2)	1101/1806 (61.0)	984/1735 (56.7)	6610/9440 (70.0)
Hypertension	2694/4729 (57.0)	84 677/128 933 (65.7)	2402/3605 (66.6)	36 938/86 081 (42.9)
Diabetes	667/4729 (14.1)	33 651/128 933 (26.1)	722/3605 (20.0)	12 508/85 364 (14.7)
Dyslipidemia	146/3934 (3.7)	314/2772 (11.3)	106/2250 (4.7)^b^	385/60 795 (0.6)
Ischemic heart disease	406/3476 (11.7)	265/1504 (17.6)	106/447 (23.7)	318/1635 (19.4)
Peripheral vascular disease	83/1868 (4.5)	82/4624 (1.8)	46/737 (6.3)^b^	491/8973 (5.5)
Atrial fibrillation	360/3934 (9.2)	20 260/130 139 (15.6)	279/1987 (14.0)	11 266/80 757 (14)
Carotid stenosis	879/3566 (24.7)	165/1086 (15.2)	271/1419 (19.1)	2655/53 905 (4.9)
Prior TIA	436/2188 (19.9)	214/1349 (15.9)	164/880 (18.6)	3225/17 332 (18.6)
Prior stroke	227/3309 (6.9)	14 784/126 332 (11.7)	311/1674 (18.6)	6486/30 880 (21.0)
Prior TIA or stroke	663/3327 (19.9)	15 293/127 629 (12.0)	738/2680 (27.5)	7467/18 240 (40.9)
Smoking	772/3633 (21.2)	8134/124 447 (6.5)	488/2423 (20.2)^b^	10 689/80 031 (13.4)

Abbreviations: ABCD^2^, age, blood pressure, clinical features, duration of transient ischemic attack, diabetes; TIA, transient ischemic attack.

^a^ All comparisons between the TIA clinic cohort and inpatient cohort are significantly different (*P* < .001). Unless otherwise noted, all comparisons between the TIA clinic cohort and emergency department cohort are significantly different (*P* < .001).

^b^ Indicates *P* > .05 in comparison between TIA clinic cohort and emergency department cohort.

In 15 cohorts (5636 patients; mean [SD] age, 65.7 [3.9] years; 2291 of 4513 [50.8%] men) acute care was delivered in TIA clinics (A. Mowla, MD, unpublished data, 2020).^9,29,30,31,32,51,52,53,54,55,56,57,58,59^ Among the inpatients (33 cohorts; 130 139 patients; mean [SD] age, 78.3 [4.0] years; 49 458 of 128 745 [38.4%] men), 21 cohorts (125 719 patients) received care in medical-surgical units (A. Mowla, MD, unpublished data, 2020),^9,15,27,32,38,39,40,41,51,52,53,55,58,59,64,65,79,81,83,84^ 5 cohorts (2487 patients) in stroke units,^49,63,79,80,92^ and 7 cohorts (1933 patients) in observation units.^48,65,66,82,83,84,95^ In 16 cohorts (3605 patients; mean [SD] age, 68.9 [3.9] years; 1596 of 3046 [52.4%] men), the acute care was offered at the ED (A. Mowla, MD, unpublished data, 2020).^15,33,34,35,36,37,40,61,67,68,81,85,87,93^ The setting of care was not fully described or the study included the patients who received treatment in various care settings and multiple centers in 37 cohorts (87 303 patients; mean [SD] age, 70.8 [3.8] years, 43 495 of 87 303 [49.8%] men).^27,42,43,44,45,46,47,50,51,53,59,60,62,69,70,71,72,73,74,75,76,77,78,79,81,86,88,89,90,92,94^ Eight studies^9,32,51,52,53,58,59^ (and A. Mowla, MD, unpublished data, 2020) provided the outcome of the patients in both inpatient and TIA clinic cohorts. The risk of subsequent stroke was reported for 35 356 patients within 2 days, 36 134 patients within 7 days, 142 185 patients within 30 days, and 94 731 patients within 90 days.

Among the patients who were referred to TIA clinics, 3 studies^29,52,56^ reported a clinic no-show rate of 36.0% (447 of 1241 referred patients with suspected cerebral ischemia). The evaluation window at the TIA clinics was within 24 hours in 101 patients,^29^ within 72 hours in 22 patients,^32^ within 1 week in 828 patients,^51,52,54,59^ and within 2 weeks among 857 patients.^31,56^ One study^58^ with 982 patients determined the appropriate interval according to ABCD^2^ score. Three studies^51,52,54^ reported the complication risk while the patients were waiting to be seen in the outpatient clinic after being discharged from the ED. This risk was zero in 2 studies (165 patients)^53,59^ and 0.6% in one study (1 of 157).^51^

Final diagnosis of TIA and mIS was made in 2895 out 4302 patients (67.3%) evaluated in the TIA clinics and 689 of 1055 patients (65.3%) of inpatients (*P* = .22). ABCD^2^ score of 4 or greater was reported in 1933 of 3703 patients (52.2%) treated at a TIA clinic and 1101 of 1806 patients (61.0%) treated as inpatients (*P* < .001). Although patients treated at a TIA clinic had lower ABCD^2^ scores compared to inpatients (TIA clinic patients with ABCD^2^ score >3, 1933 of 3703 [52.2%]; inpatients with ABCD^2^ score >3, 1101 of 1806 [61.0%]) (Table), this score did not seem to affect the risk estimation under different setting of care when we considered all cohorts or when we estimated the risk within each evaluation time (eTable 2 in the Supplement). More patients treated in TIA clinics had carotid stenosis than those treated as inpatients (879 of 3566 [24.7%] vs 214 of 1349 [15.9%]).

### Outcome of Meta-analyses

**Figure 2.**
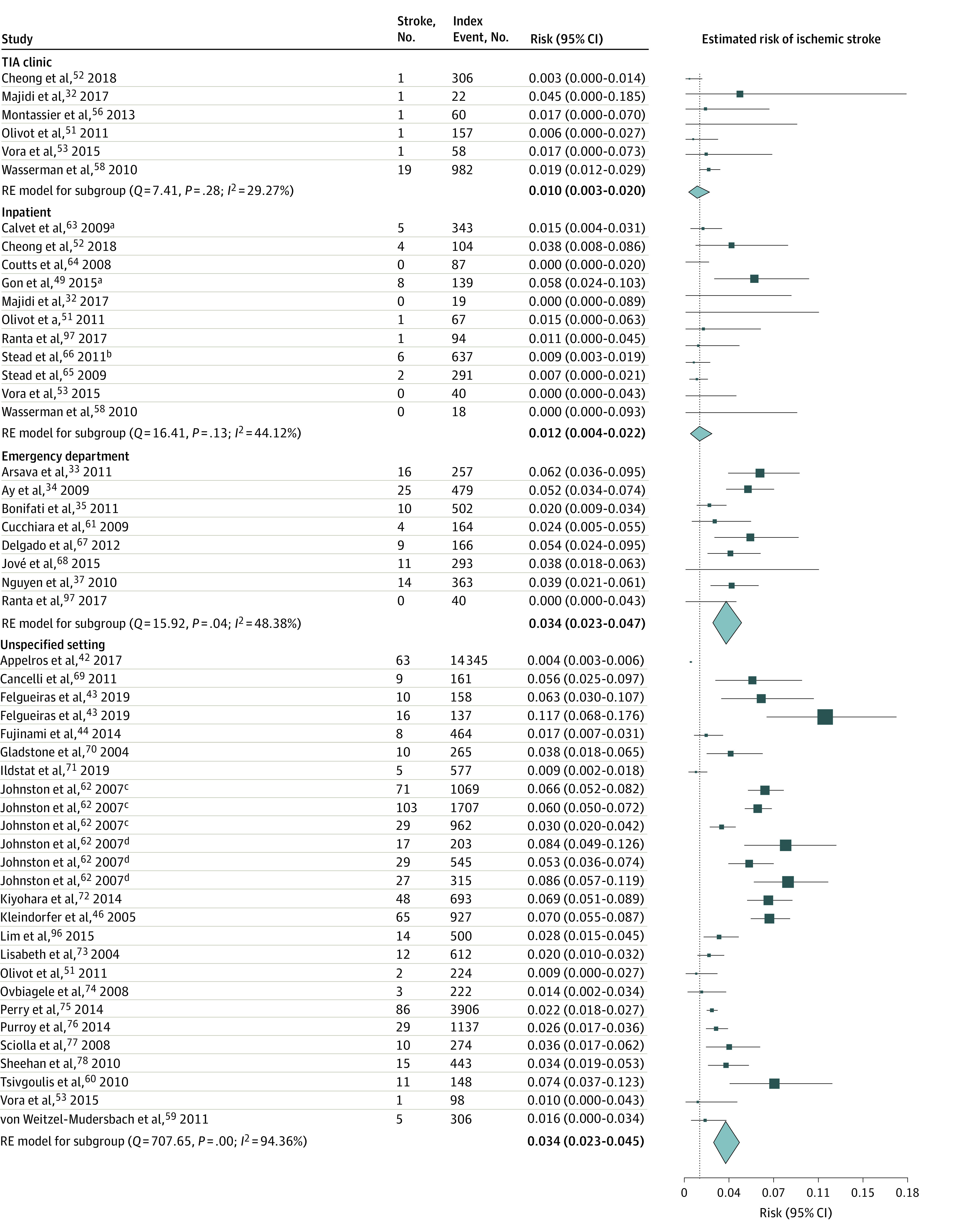
Risk of Subsequent Ischemic Stroke Within 7 Days of the Index Event by Care Setting The risk estimate for inpatients was considered as the reference line. ^a^Indicates the stroke unit. ^b^Indicates the observation unit. ^c^Kaiser Permanente Medical Care Program. ^d^Oxfordshire Community Stroke Project.

**Figure 3.**
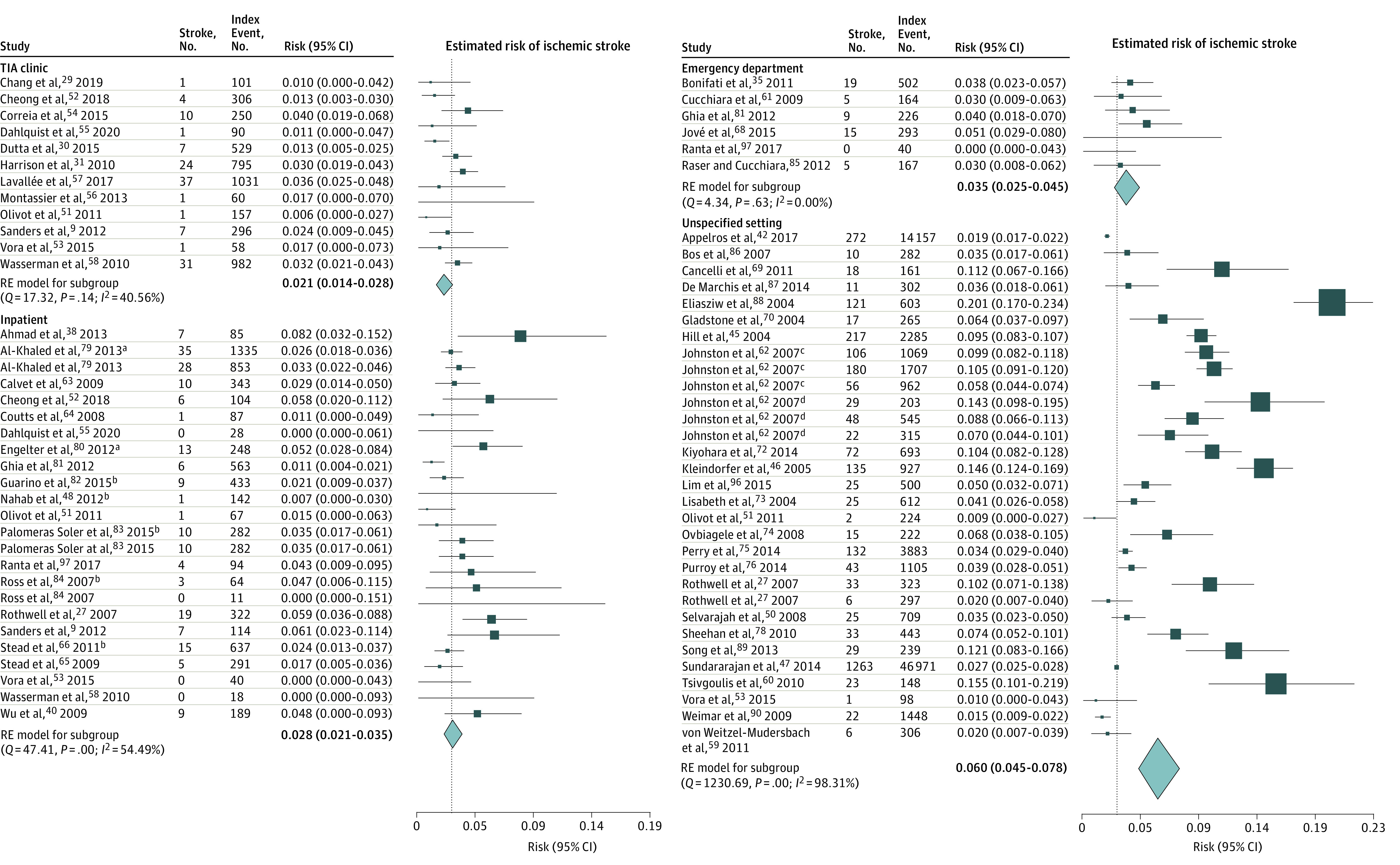
Risk of Subsequent Ischemic Stroke Within 90 Days of the Index Event by Care Setting The risk estimate for inpatients was considered as the reference line. ^a^Indicates the stroke unit. ^b^Indicates the observation unit. ^c^Kaiser Permanente Medical Care Program. ^d^Oxfordshire Community Stroke Project.

As presented in the eTable 4 in the Supplement, the difference among estimated risk of subsequent stroke measured by seven estimators (DL, HE, HS, ML, REML, SJ, and EB) was negligible. The forest plots based on the REML estimator^43,60,61,62,63,64,65,66,67,68,69,70,71,72,73,74,75,76,77,78,79,80,81,82,83,84,85,86,87,88,89,90,96,98,99,100,101,102,103,104,105,106,107,108,109,110,111,112,113,114,115,116,117,118,119,120,121,122,123^ are shown Figure 2 and Figure 3 and eFigure 2 and eFigure 3 in the Supplement. Among the patients who were treated in a TIA clinic, the risk of subsequent stroke following a TIA or mIS was 0.3% (95% CI, 0.0%-1.2%) within 2 days, 1.0% (95% CI, 0.3%-2.0%) within 7 days, 1.3% (95% CI, 0.4%-2.6%) within 30 days, and 2.1% (95% CI, 1.4%-2.8%) within 90 days. Among the patients who were treated as inpatients, the risk of subsequent stroke was 0.5% (95% CI, 0.1%-1.1%) within 2 days, 1.2% (95% CI, 0.4%-2.2%) within 7 days, 1.6% (95% CI, 0.6%-3.1%) within 30 days, and 2.8% (95% CI, 2.1%-3.5%) within 90 days. At the EDs, the risk was 1.9% (95% CI, 1.2%-2.7%) within 2 days, 3.4% (95% CI, 2.3%-4.7%) within 7 days, 3.5% (95% CI, 1.5%-6.3%) within 30 days, and 3.5% (95% CI, 2.5%-4.5%) within 90 days. In the cohort of patients from unspecified settings the risk of subsequent stroke was reported under considerable heterogeneity in all intervals as 2.2% (95% CI, 1.3%-3.1%) within 2 days, 3.4% (95% CI, 2.3%-4.5%) within 7 days, 4.2% (95% CI, 2.8%-5.9%) within 30 days, and 6.0% (95% CI, 4.5%-7.8%) within 90 days.

Comparing the subsequent stroke risk estimates in the cohort of patients treated in the TIA clinics vs inpatient settings did not reveal a significant difference in any of the 4 evaluation intervals (eTable 5 in the Supplement). In comparison with patients referred to TIA clinics and hospitalized patients, those who received care in the ED had a significantly higher risk of subsequent stroke at 2 and 7 days (for inpatients) and 2, 7, and 90 days (for patients referred to TIA clinics) (eTable 5 in the Supplement).

In the sensitivity analyses in which only prospective cohorts recruited after 2000 were included (eFigures 4-7 and eTable 5 in the Supplement), we did not find significant differences in risk among patients treated in the TIA clinics and inpatients at 2 days (0.2% [95% CI, 0-1.0%] vs 0.3% [95% CI, 0-0.8%] among inpatients; *P* = .94, *I*^2^ < 0.001) (eFigure 4 in the Supplement), 7 days (0.8% [95% CI, 0.2%-1.8%] vs 0.7% [95% CI, 0.3%-1.3%] among inpatients; *P* = .81, *I*^2^ < 0.001) (eFigure 5 in the Supplement), 30 days (1.3% [95% CI, 0.4%-2.5%] vs 1.3% [0.3%-2.7%] among inpatients; *P* > .99; *I*^2^ < 0.001) (eFigure 6 in the Supplement), and 90 days (2.2% [95% CI, 1.5%-3.0%] vs 2.6% [95% CI, 1.9%-3.3%] among inpatients; *P* = .46; *I*^2^ < 0.001) (eFigure 7 in the Supplement).

### Quality Assessment of the Included Cohorts

#### Publication Bias Assessment

Funnel plots presenting the publication bias of studies within 2, 7, 30, and 90 days under each setting of care (TIA clinic, inpatient, ED, and unspecified setting) are available in eFigure 1 in the Supplement. Neither the Begg-Mazumdar rank correlation, Kendall τ^2^ statistic, or the Egger bias test could detect publication bias among included cohorts (eTable 6 in the Supplement).

#### Risk-of-Bias Assessment

eTable 7 in the Supplement summarizes the results of the risk of bias assessment according to ROBINS-E. Among the 63 cohorts with specified settings (ie, TIA clinic, inpatient, and ED) 59 cohorts (90.6%) had low risk of bias,^9,13,30,31,32,33,34,35,36,37,38,40,41,42,43,44,45,48,49,50,51,53,54,55,56,57,58,59,60,61,62,63,64,67,68,69,70,71,72,73,74,75,76,77,79,81,82,83,84,85,86,87,88,90,91,92,93,94,95,96^ and 4 cohorts (9.4%) had a moderate overall risk of bias.^39,46,52,80^

#### Heterogeneity Assessment

We considered 7 different estimators (DL, HS, HE, ML, REML, SJ, and EB) to assess the heterogeneity in the risk of stroke after TIA within 2, 7, 30, and 90 days under each care setting (eTable 4 in the Supplement). Overall, the HE resulted in lower *I*^2^, and the SJ estimator resulted in higher *I*^2^ values in comparison with other estimators. The heterogeneity among the cohort of patients treated in TIA clinics was minimal, regardless of the estimator or evaluation time.

## Discussion

Comparing the risk of subsequent stroke at 2, 7, 30, and 90 days after a TIA or mIS suggested that offering rapid management at TIA clinics is not inferior to inpatient care models. Among the identified cohort, patients who received care at TIA clinics were younger, had a higher rate of carotid stenosis, but a lower ABCD^2^ score.

Our results also suggest an increased risk of subsequent stroke in patients who were treated and discharged from ED without assigned follow-up care. Previous studies have reported that patients with TIA who were discharged from ED were less likely to receive guideline-concordant care and underwent fewer timely brain and carotid imaging, monitoring for arrhythmia, and administration of preventive medications such as antithrombotic, antihypertensive, and lipid-lowering agents.^98,99^ The risk of recurrence can be stratified by the clinical scales (such as ABCD^2^) and risk factor profiles^37,60,61,100,101,102,103,104,105,106^; however, many practitioners, especially in community hospitals, rely on a one-size-fits-all approach.^5,107,108^

However, there is growing evidence that suggests TIA clinics can be considered an alternative to hospitalization.^1,9,10,12,13,14^ Despite the very different structures of risk stratification and patient selection, referral patterns, and diagnostic and therapeutic protocols in these TIA clinic models, the risk of cerebral ischemia in patients treated at a TIA clinic did not exceed those treated in an inpatient setting.^32,51,53,55,58,59^

Many practice guidelines also endorse outpatient TIA management and recommend hospitalization of high-risk patients. The American Heart/American Stroke Association (AHA/ASA) guideline^21^ recommends an urgent evaluation and hospitalization of TIA patients if they present within 72 hours and have an ABCD^2^ score of 3 or greater.^62^ The Australian National Stroke Foundation includes a set of high-risk indicators beside the ABCD^2^ scoring into the triaging criteria. This guideline recommends urgent and comprehensive management of TIA by use of a local TIA pathway covering primary care, emergency, and stroke specialist teams within locally available resources.^109^ The United Kingdom national guideline^110^ and Canadian Stroke Best Practice Recommendations^111^ consider the time elapsed from symptoms onset and risk of early recurrence. The United Kingdom guideline recognizes outpatient clinics to provide care for TIA patients. The Canadian guideline also states that while high-risk patients should be seen within 24 hours, providing care for other patients can be slightly delayed based on their risk scoring.

### Challenges of TIA Outpatient Care

Differentiation between vascular and nonvascular causes of TIA-like presentations is challenging, especially for nonneurologists.^2,112,113,114^ Our previous study^113^ and others^112,115,116,117,118^ indicate that the percentage of TIA misdiagnosis can be as high as 60% in EDs and primary care offices. Approximately half of the patients with clinical presentations of cerebral ischemia have the final diagnosis of a TIA mimic, and many of them may present with a high ABCD^2^ score.^100,119^ A meta-analysis found that 20% of patients with an ABCD^2^ score of less than 4 had atrial fibrillation or more than 50% had carotid stenosis.^100^ One step toward the timely and efficient management of TIA is reducing diagnostic errors by providing education and using advanced diagnostic and management-assistive tools by leveraging electronic health records and advanced predictive tools.^15,120,121^ Moreover, providing timely outpatient care to TIA patients is challenging. It is critically important to understand the potential delays through the clinical care pathways from symptom onset to a specialist.

### Value of TIA Clinic

Although several studies have found TIA clinics could substantially reduce the cost of care,^9,122^ evaluation of the cost-effectiveness of TIA clinics remains limited in the literature. Beyond clinical management, the benefits of TIA clinics could also include a more accurate diagnosis for patients with suspected TIA compared with inpatient and ED settings, fast-track access to specialists, and appropriate patient education and follow-up,^123^ which could depend on the infrastructure and resources of the existing health service system.

### Strengths and Limitations

In this study, we attempted to systematically review the available literature on TIA care models. However, our study has several limitations. We did not consider the details of diagnostic and therapeutic measures in each care setting, variability in the definition of TIA or minor stroke, and the health care system and referral rules in each country in the meta-analyses. Although data were sparse in terms of patients with ABCD^2^ scores of less than 4, we observed that patients with lower ABCD^2^ scores were more likely to be referred to outpatient clinics, and hospitals considered different thresholds of cerebral ischemia severity for discharging the patients. Although we were able to calculate the rate of TIA overdiagnosis in TIA clinic and inpatient settings, we did not have enough detailed information to calculate the outcome among patients who had the final diagnosis of TIA or mIS. Nevertheless, the rates of overdiagnosis were similar between inpatient and TIA clinic cohorts. In addition, we observed a discrepancy in the size of patient cohorts under each setting of care, which can explain some of the residual confounding. These assumptions may affect the conclusion regarding the safety of TA clinics compared with other care settings. However, they may propose a practical algorithm for the triage of patients with TIA and safely offer outpatient care for those at a lower risk. We were not able to further clarify and subcategorize patients in unspecified settings. However, by including the cohorts from national or multicentric registries (unspecified settings), we highlighted the variability of outcomes among patients treated in the absence of defined care protocols.

## Conclusions

This systematic review and meta-analysis of the outcome of 226 683 patients who experienced a transient ischemic stroke or minor stroke suggest the risk of subsequent stroke among patients who were evaluated in a TIA clinic was not higher than that among those hospitalized. Patients who were treated in EDs without further follow-up had a higher risk of subsequent stroke.

## Notes

Supplement. **eAppendix.** Search Protocols **eTable 1.** Mixed-Effect Models Considering Different Possible Moderators **eTable 2.** Mixed-Effect Model Considering ABCD^2^ Scores **eTable 3.** Excluded Studies **eTable 4.** Heterogeneity and Risk of Stroke Assessment Based on Different Estimators **eTable 5.** Comparison of Risk Estimates **eTable 6.** Publication Bias Assessment **eTable 7.** Risk-of-Bias Assessment Based on ROBINS-E Tool **eFigure 1.** Funnel Plots **eFigure 2.** Risk of Subsequent Ischemic Stroke Within 2 Days **eFigure 3.** Risk of Subsequent Ischemic Stroke Within 30 Days **eFigure 4.** Sensitivity Analysis: Risk of Subsequent Ischemic Stroke Within 2 Days **eFigure 5.** Sensitivity Analysis: Risk of Subsequent Ischemic Stroke Within 7 Days **eFigure 6.** Sensitivity Analysis: Risk of Subsequent Ischemic Stroke Within 30 Days **eFigure 7.** Sensitivity Analysis: Risk of Subsequent Ischemic Stroke Within 90 Days
eReferences.
