# Supplementary material for: Risk of Subsequent Stroke Among Patients Receiving Outpatient vs Inpatient Care for Transient Ischemic Attack: A Systematic Review and Meta-analysis
Source: JAMA Netw Open. 2022 Jan 5;5(1):e2136644. doi: 10.1001/jamanetworkopen.2021.36644 (PMC8733831; doi:10.1001/jamanetworkopen.2021.36644)
Supplement: Supplement. — eAppendix. Search Protocols eTable 1. Mixed-Effect Models Considering Different Possible Moderators eTable 2. Mixed-Effect Model Considering ABCD2 Scores eTable 3. Excluded Studies eTable 4. Heterogeneity and Risk of Stroke Assessment Based on Different Estimators eTable 5. Comparison of Risk Estimates eTable 6. Publication Bias Assessment eTable 7. Risk-of-Bias Assessment Based on ROBINS-E Tool eFigure 1. Funnel Plots eFigure 2. Risk of Subsequent Ischemic Stroke Within 2 Days eFigure 3. Risk of Subsequent Ischemic Stroke Within 30 Days eFigure 4. Sensitivity Analysis: Risk of Subsequent Ischemic Stroke Within 2 Days eFigure 5. Sensitivity Analysis: Risk of Subsequent Ischemic Stroke Within 7 Days eFigure 6. Sensitivity Analysis: Risk of Subsequent Ischemic Stroke Within 30 Days eFigure 7. Sensitivity Analysis: Risk of Subsequent Ischemic Stroke Within 90 Days eReferences. [file jamanetwopen-e2136644-s001.pdf]

Supplemental Online Content

Shahjouei S, Li J, Koza E, et al. Risk of subsequent stroke among patients receiving outpatient vs inpatient care for transient ischemic attack: a systematic review and meta-analysis. *JAMA Netw Open.* 2022;5(1):e2136644. doi:10.1001/jamanetworkopen.2021.36644

eAppendix. Search Protocols

eTable 1. Mixed-Effect Models Considering Different Possible Moderators

eTable 2. Mixed-Effect Model Considering ABCD^2 Scores

eTable 3. Excluded Studies

eTable 4. Heterogeneity and Risk of Stroke Assessment Based on Different Estimators

eTable 5. Comparison of Risk Estimates

eTable 6. Publication Bias Assessment

eTable 7. Risk-of-Bias Assessment Based on ROBINS-E Tool

eFigure 1. Funnel Plots

eFigure 2. Risk of Subsequent Ischemic Stroke Within 2 Days

eFigure 3. Risk of Subsequent Ischemic Stroke Within 30 Days

eFigure 4. Sensitivity Analysis: Risk of Subsequent Ischemic Stroke Within 2 Days

eFigure 5. Sensitivity Analysis: Risk of Subsequent Ischemic Stroke Within 7 Days

eFigure 6. Sensitivity Analysis: Risk of Subsequent Ischemic Stroke Within 30 Days

eFigure 7. Sensitivity Analysis: Risk of Subsequent Ischemic Stroke Within 90 Days

eReferences.

This supplemental material has been provided by the authors to give readers additional information about their work.

© 2022 Shahjouei S et al. *JAMA Network Open.*

eAppendix. Search Protocols

1. Medline via Ovid
2. and All EBM reviews via Ovid

Statement 1 AND statement 2 AND statement 3 AND statement 4

Statement 1: Transient Ischemic Attack

(exp Ischemic Attack, Transient/) or (transient isch?emic attack$.mp.) or (TIA.mp.) or (transient neurologic$ attack$.mp.) or (TNA.mp.) or (mini-stroke.mp.) or (minor adj2 stroke.mp.) or (transient focal neurologic$ symptom$.mp.) or (transient focal neurologic$ dysfunction$.mp.)

Statement 2: Care Setting - Format search as Part 1 or Part 2 or Part 3

Part 1
(exp ambulatory care facilities/) or (ambulatory care facilit$.mp.) or (clinic$.mp.) or (exp Ambulatory Care/) or (exp Outpatient Clinics, Hospital/) or (exp Outpatients/) or (exp Mobile Health Units/) or (exp Outpatients/) or (exp Outpatient Clinics, Hospital/) or (exp Emergency Service, Hospital/) or (exp Monitoring, Ambulatory/) or (exp Self-Care Units/) or (exp Clinical Observation Units/) or (exp Secondary Prevention/) or ((initial or urgent or rapid or semi?urgent or emergency) adj3 (evaluation$ or assessment$ or care))

Part 2
(initial adj3 management$.mp) or (primary adj3 management$.mp.) or ((rapid or urgent or semi?urgent) adj3 diagnos$s.mp) or (model adj2 care$.mp.) or (exp Triage/) or (post?TIA care.mp.) or (TIA adj2 consult$.mp.) or (stroke adj2 specialist$.mp.) or (stroke adj2 clinician$) or (service location.mp.) or (service modification$.mp.) or (service configuration.mp.) or (prevention$ clinic$.mp) or (acute care.mp.) or (exp subacute care/) or (acute adj2 management.mp.) or (exp Disease Management/)

Part 3
(care resource$.mp) or (structure$ adj2 program$.mp.) or (exp referral and consultation/) or (disposition adj2 TIA.mp.) or (disposition adj2 transient isch?emic attack$.mp.) or (disposition adj2 ED.mp.) or (disposition adj2 emergency department$.mp.) or (exp caregivers/) or (care?giv$.mp.) or (care?giver$ effort$.mp) or (care?giver$ guide$.mp.) or (care?giver$ practice$.mp.) or (care?giver$ team$.mp.) or (pathway$.mp) or (pre?hospital$.mp.) or (ambu$lance$.mp.) or (para?medic$.mp.) or (risk$ stratification$.mp.)

Statement 3: Outcomes - Subsequent Cerebral Ischemia, LOS, mortality, readmission
(exp cerebrovascular disorders/) or (Hypoxia-Ischemia, Brain/) or (exp Brain Ischemia/) or (exp stroke/) or (exp cerebral infarction/) or (exp Brain Infarction/) or (Brain adj2 Infarct$.mp.) or (endovascular.mp.) or (cerebr$ artr$ occlusion$.mp.) or (cerebr$ vascul$infarct$.mp) or (cerebrovasc$ infarct$.mp.) or (cerebr$ vasc$ event$.mp.) or (cerebral artr$ infarct$.mp.) or (cerebrovasc$ event$.mp.) or (cerebr$ vasc$ accident$.mp.) or (cerebrovasc$ accident$.mp.) or (cva.mp.) or (cerebr$ infarct$.mp) or (cerebr$ isch?emia$.mp.) or (medullary infarct$.mp.) or (subsequent adj2 stroke.mp.) or (recur$ adj2 stroke.mp) or (minor adj2 stroke) or (recur$ adj2 isch?emia.mp.) or (length adj3 stay) or (exp mortality/) or (exp death/) or (re?admission$ adj2 stroke) or exp patient readmission/ or exp length of stay/ or (exp Survival/) or survival.mp
Statement 4: Methods of measuring/assessing – Format search as: Part 1 or Part 2 or Part 3 or Part 4 or Part 5 or Part 6 or Part 7

Part 1
(evaluation studies/) or (evaluation studies as topic/) or ("evaluation studies".pt.) or ("validation studies".pt.) or (exp evaluation studies as topic/) or (Pilot projects/) or (program evaluation/) or (validation studies as topic/) or (Intervention Studies/) or (((pre- adj5 post-) or (pretest adj5 posttest)) and (program$ adj6 evaluat$))

Part 2

comparative study.pt or (before adj3 after) or prognos$ or prediction$ or predictor$ or (disease adj3 (course or progression))

Part 3
(modifier$1) or (mediator$1) or (natural histor$) or ((precision or individuali?ed or personali?ed or stratified or systems) adj3 medicine) or (exp models, statistical/) or cohort$ or (follow-up$ adj3 stud$) or case?control or case?controlled

Part 4
((prospective$ or longitudinal or observation$) adj3 stud$) or time series.mp or (exp incidence/) or (exp follow-up studies/) or registries/ or (systematic review) or (structured review.ti.) or (exp Clinical Trials as Topic/) or (clinical trial$) or (randomised controlled trial$) or (randomised trial$) or (workgroup$) or (Practice Guideline/) or (standard$ adj3 reporting)

Part 5
congresses.pt or Delphi Technique/ or (consensus development conference.pt) or (priorit$ symptom$) or (endpoint determination/) or (consensus development conference/) or (patient participation/) or consensus.mp or workshop or (Consensus Development Conferences, NIH as Topic/) or outcome$ or (end point$) or (core adj3 set) or (treatment emergent problem$) or (exp outcome Assessment Health Care/) or (Treatment Outcome/) or (Quality of Life/) or (patient$ adj2 perspective$)

Part 6
(clinical trial) adj 4 design$ or (exp patient outcome assessment/) or (exp delivery of health care/) or (exp epidemiologic studies/) or (exp clinical pathway/) or (exp clinical protocol/) or (exp consensus/) or (exp consensus development conference/) or (exp consensus development conferences as topic/) or (critical pathways/) or (exp guideline/) or (guidelines as topic/) or (exp practice guideline/) or (practice guidelines as topic/) or (health planning guidelines/) or (exp treatment guidelines/) or (guideline or practice guideline or consensus development conference or consensus development conference, NIH).pt

Part 7
position statement$ or policy statement$ or practice parameter$ or best practice$ or standards or guideline$ or consensus$ or ((critical or clinical or practice) adj2 (path or paths or pathway or pathways or protocol$)) or recommendation$ or (care adj2 (standard or path or paths or pathway or pathways or map or maps or plan or plans)) or (algorithm$ adj4 (screening or examination or test or tested or testing or assessment$ or diagnos?s or pharmacotherap$ or chemotherap$ or chemotreatment$ or therap$ or treatment$ or intervention$)) or (predict$.mp.) or (scor$.tw.)

3. Web of Science via Clarivate Analytics

Refined by: WEB OF SCIENCE CATEGORIES: (CLINICAL NEUROLOGY OR PERIPHERAL VASCULAR DISEASE OR NEUROSCIENCES OR RADIOLOGY NUCLEAR MEDICINE MEDICAL IMAGING OR NEUROIMAGING OR REHABILITATION)

Statement 1 AND Statement 2 AND Statement 3

Statement 1: Transient Ischemic Attack

(“Transient Ischemic Attack”) OR (“transient isch?emic attack*”) OR (TIA) OR (“transient neurologic* attack*”) OR (TNA) OR (mini?stroke) OR (minor stroke) OR (“transient focal neurologic* symptom*”) OR (“transient focal neurologic* dysfunction*”)

Statement 2: Care Setting - Part 1 OR Part 2 OR Part 3

Part 1
(“ambulatory care facilit*”) OR (clinic*) OR (“Ambulatory Care”) OR (“Outpatient Clinic*”) OR (Outpatients) OR (“Mobile Health Unit*”) OR (“Emergency Service”) OR (“Ambulatory Monitoring”) OR (“Clinical Observation* Unit*”) OR ((initial OR urgent OR rapid OR semi?urgent OR emergency) AND (evaluation* OR assessment* OR care))

Part 2

© 2022 Shahjouei S et al. *JAMA Network Open.*

(initial management*) OR (primary management*) OR (rapid diagnosis) OR (urgent diagnosis) OR (semi?urgent diagnosis) OR (Triage) OR ("post?TIA care") OR ("prevention* clinic*") OR ((acute OR sub?acute OR Urgent OR semi?urgent OR rapid OR fast OR initial) AND (care OR management)) OR ("Disease Management")

Part 3
("care resource*") OR (structure* W/2 program*) OR (referral) OR (consultation) OR (disposition W/2 TIA) OR (disposition AND ("transient isch?emic attack*" OR ED OR "emergency department*")) OR (caregivers) OR (pathway*) OR (pre?hospital*) OR (ambu*lance*) OR (para?medic*) OR (risk* stratification*)

Statement 3: Outcomes - Subsequent Cerebral Ischemia, LOS, mortality, readmission

("cerebrovascular disorder*") OR ("Brain Ischemia") OR ("ischemic brain injury") OR (stroke) OR ("cerebral infarction") OR ("Brain Infarction*") OR ("cerebr* artr* occlusion*") OR ("cerebr* vascul*infarct*") OR ("cerebrovasc* infarct*") OR ("cerebr* vasc* event*") OR ("cerebral artr* infarct*") OR ("cerebrovasc* event*") OR ("cerebr* vasc* accident*") OR ("cerebrovasc* accident*") OR (cva) OR ("cerebr* infarct*") OR ("cerebr* isch?emia*") OR ("medullary infarct*") OR ((subsequent OR recur* OR minor) AND (stroke OR isch?emia)) OR (length ? stay) OR (mortality) OR (death) OR (re?admission* ? stroke) OR ("patient readmission") OR ("length of stay") OR (Survival)

4. EMBASE

Statement 1 AND statement 2 AND statement 3 AND statement 4

Statement 1: Transient Ischemic Attack

transient AND ischemic AND attack OR (transient AND ischemic AND attack.mp.) OR tia.mp. OR tna.mp. OR 'mini stroke.mp.' OR (minor AND stroke) OR (transient AND focal AND neurologic AND symptom.mp.) OR (transient AND focal AND neurologic AND dysfunction.mp.)

Statement 2: Care Setting- Format search as Part 1 or Part 2 or Part 3

Part 1
(exp ambulatory care facilities/) or (ambulatory care facilit$.mp.) or (clinic$.mp.) or (exp Ambulatory Care/) or (exp Outpatient Clinics) or (exp Outpatients/) or (exp Outpatients/) or (exp Outpatient Clinics/) or (exp Emergency Service/) or (exp Clinical Observation Units/) or ((initial or urgent or rapid or semi?urgent or emergency) adj3 (evaluation$ or assessment$ or care))

Part 2
(initial adj3 management$.mp) or (primary adj3 management$.mp.) or ((rapid or urgent or semi?urgent) adj3 diagnos$s.mp) or (model adj2 care$.mp.) or (exp Triage/) or (post?TIA care.mp.) or (TIA adj2 consult$.mp.) or (stroke adj2 specialist$.mp.) or (stroke adj2 clinician$) or (service location.mp.) or (service modification$.mp.) or (service configuration.mp.) or (prevention$ clinic$.mp) or (acute care.mp.) or (exp subacute care/) or (acute adj2 management.mp.) or (exp Disease Management/)

Part 3
(care resource$.mp) or (structure$ adj2 program$.mp.) or (exp referral and consultation/) or (disposition adj2 TIA.mp.) or (disposition adj2 transient isch?emic attack$.mp.) or (disposition adj2 ED.mp.) or (disposition adj2 emergency department$.mp.) or (exp caregivers/) or (care?giv$.mp.) or (care?giver$ effort$.mp) or (care?giver$ guide$.mp.) or (care?giver$ practice$.mp.) or (care?giver$ team$.mp.) or (pathway$.mp) or (pre?hospital$.mp.) or (ambu$lance$.mp.) or (para?medic$.mp.) or (risk$ stratification$.mp.)

Statement 3: Outcomes - Subsequent Cerebral Ischemia, LOS, mortality, readmission

(exp cerebrovascular disorders/) or (exp Brain Ischemia/) or (exp stroke/) or (exp cerebral infarction/) or (exp Brain Infarction/) or (Brain adj2 Infarct$.mp.) or (endovascular.mp.) or (cerebr$ artr$ occlusion$.mp.) or (cerebr$ vascul$infarct$.mp) or (cerebrovasc$ infarct$.mp.) or (cerebr$ vasc$ event$.mp.) or (cerebral artr$ infarct$.mp.) or (cerebrovasc$ event$.mp.) or (cerebr$ vasc$ accident$.mp.) or (cerebrovasc$ accident$.mp.) or (cva.mp.) or (cerebr$

infarct$.mp) or (cerebr$ isch?emia$.mp.) or (medullary infarct$.mp.) or (subsequent adj2 stroke.mp.) or (recur$ adj2 stroke.mp) or (minor adj2 stroke) or (recur$ adj2 isch?emia.mp.) or (length adj3 stay) or (exp mortality/) or (exp death/) or (re?admission$ adj2 stroke) or exp patient readmission/ or exp length of stay/ or (exp Survival/) or survival.mp

Statement 4: Methods of measuring/assessing – Format search as: Part 1 or Part 2 or Part 3 or Part 4 or Part 5 or Part 6 or Part 7

Part 1

(evaluation studies/) or (evaluation studies as topic/) or ("evaluation studies".pt.) or ("validation studies".pt.) or (exp evaluation studies as topic/) or (Pilot projects/) or (program evaluation/) or (validation studies as topic/) or (Intervention Studies/) or (((pre- adj5 post-) or (pretest adj5 posttest)) and (program$ adj6 evaluat$))

Part 2

comparative study.pt or (before adj3 after) or prognos$ or prediction$ or predictor$ or (disease adj3 (course or progression))

Part 3

(modifier$1) or (mediator$1) or (natural histor$) or ((precision or individuali?ed or personali?ed or stratified or systems) adj3 medicine) or cohort$ or (follow-up$ adj3 stud$) or case?control or case?controlled

Part 4

((prospective$ or longitudinal or observation$) adj3 stud$) or time series.mp or (exp incidence/) or (exp follow-up studies/) or registries/ or (systematic review) or (structured review.ti.) or (exp Clinical Trials as Topic/) or (clinical trial$) or (randomised controlled trial$) or (randomised trial$) or (workgroup$) or (Practice Guideline/) or (standard$ adj3 reporting)

Part 5

congresses.pt or Delphi Technique/ or (consensus development conference.pt) or (priorit$ symptom$) or (endpoint determination/) or (consensus development conference/) or (patient participation/) or consensus.mp or workshop or (Consensus Development Conferences, NIH as Topic/) or outcome$ or (end point$) or (core adj3 set) or (treatment emergent problem$) or (exp outcome Assessment Health Care/) or (Treatment Outcome/) or (Quality of Life/) or (patient$ adj2 perspective$)

Part 6

(clinical trial) adj 4 design$ or (exp patient outcome assessment/) or (exp delivery of health care/) or (exp epidemiologic studies/) or (exp clinical pathway/) or (exp clinical protocol/) or (exp consensus/) or (exp consensus development conference/) or (exp consensus development conferences as topic/) or (critical pathways/) or (exp guideline/) or (guidelines as topic/) or (exp practice guideline/) or (practice guidelines as topic/) or (health planning guidelines/) or (exp treatment guidelines/) or (guideline or practice guideline or consensus development conference or consensus development conference, NIH).pt

Part 7

position statement$ or policy statement$ or practice parameter$ or best practice$ or standards or guideline$ or consensus$ or ((critical or clinical or practice) adj2 (path or paths or pathway or pathways or protocol$)) or recommendation$ or (care adj2 (standard or path or paths or pathway or pathways or map or maps or plan or plans)) or (algorithm$ adj4 (screening or examination or test or tested or testing or assessment$ or diagnos?s or pharmacotherap$ or chemotherap$ or chemotreatment$ or therap$ or treatment$ or intervention$)) or (predict$.mp.) or (scor$.tw.)

© 2022 Shahjouei S et al. *JAMA Network Open.*

5. Scopus via Ebsco

TITLE-ABS-KEY (Statement 1 AND Statement 2 AND Statement 3)

Statement 1: Transient Ischemic Attack

{Transient Ischemic Attack} OR {TIA} OR {transient neurological attack} OR {TNA} OR {minor stroke} OR {transient focal neurological symptom}

Statement 2: Care Setting

{clinic} OR {outpatient} OR {out-patient} OR {ambulatory care}

Statement 3: Outcomes
{Brain ischemia} OR {Brain infarction} OR {Stroke} OR {cerebral infarction} OR {ischemic brain injury} OR {death} OR {length of tray}

6. International Clinical Trials Registry Platform (ICTRP) search portal

("Transient ischemic attack" OR TIA OR "transient neurologic attack" OR TNA OR "mini stroke" OR "minor stroke") AND (outpatient OR "out patient" OR "clinic") AND ("cerebral infarction" OR "brain ischemia" OR stroke OR "cerebrovascular accident" OR death)

7. Clinicaltrial.gov

("Transient ischemic attack" OR TIA OR "transient neurologic attack" OR TNA OR "mini stroke" OR "minor stroke") AND (outpatient OR "out patient" OR "clinic")

8. Trip database

P: "Transient ischemic attack" OR TIA OR "transient neurologic attack" OR TNA OR "mini stroke" OR "minor stroke"

I: outpatient OR "out patient" OR "clinic"

C: inpatient OR admit OR hospitalization

O: "cerebral infarction" OR "brain ischemia" OR stroke OR "cerebrovascular accident" OR death)

9. CINAHL Complete

("Transient ischemic attack" OR TIA OR "transient neurologic attack" OR TNA OR "mini stroke" OR "minor stroke") AND (outpatient OR "out patient" OR "clinic") AND ("cerebral infarction" OR "brain ischemia" OR stroke OR "cerebrovascular accident" OR death)

© 2022 Shahjouei S et al. *JAMA Network Open.*

eTable 1. Mixed-Effect Models Considering Different Possible Moderators

Estimate / Moderator	$b Outcome Estimate	$se Standard Error	$zval Z-value	$pval P-value	$ci.lb Lower Border of Confidence Interval	$ci.ub Upper Border of Confidence Interval	$I2 Residual Heterogeneity / Unaccounted Variability	$H2 Unaccounted Variability / Sampling Variability
No Moderator	0.1818	0.0056	32.609	<.0001	0.1709	0.1927	96.03%	25.22
Evaluation Interval	0.0006	0.0001	4.4624	<.0001	0.0003	0.0009	95.15%	20.63
Study Design	-0.0242	0.0137	-1.7628	0.0779	-0.051	0.0027	95.71%	23.29
Patient Recruitment	-0.0438	0.0074	-5.898	<.0001	-0.0584	-0.0293	95.36%	21.54
ABCD2 Score	0.0015	0.0006	2.5058	0.0122	0.0003	0.0026	89.66%	9.67
Setting of Management	0.0292	0.0046	6.3724	<.0001	0.0202	0.0381	94.01%	16.69

Evaluation interval was considered within 2, 7, 30, and 90 days; Study design was defined as prospective vs. retrospective; Patient recruitment was defined a
and 2007, and after 2007; ABCD2 score was defined as percentage of patients in each cohort who had score of <4 vs. ≥4; Setting of Management was defin
and unspecified. Restricted Maximum Likelihood (REML) was considered as estimator of all models.

© 2022 Shahjouei S et al. *JAMA Network Open.*

eTable 2. Mixed-Effect Model Considering ABCD^2 Scores

Setting	Test	Parameter	Among all cohorts*	Within 2 days †	Within 7 days	Within 30 days	Within 90 days
TIA Clinic	Omnibus Test	QM	0.3430	…	0.0218	0.0957	2.8608
		df	1	…	2	2	1
		Pvalue	0.5581	…	0.8826	0.7570	0.0908
	Mixed-Effect Model	K	13	6	3	3	5
		τ2 estimator	REML	REML	REML	REML	REML
		τ2	0.0008 (SE = 0.0007)	0.0009 (SE = 0.0016)	0.0006 (SE = 0.0098)	0 (SE = 0.0008)	0.0004 (SE = 0.0008)
		I2	59.10%	37.57%	9.36%	0.00%	41.51%
		H2	2.45	1.60	1.10	1.00	1.71
		R2	0.00%	…	0.00%	0.00%	48.17%
	Model result	Zval	0.5856	4.1091	-0.1477	0.3094	1.6914
		Pvalue	0.5581	<.0001	0.8826	0.7570	0.0908
	Residual Heterogeneity	QE	26.4096	6.8685	1.1033	0.1859	4.5864
		df	11	5	1	1	2
		Pvalue	0.0056	0.2306	0.2935	0.6663	0.2047
Inpatient	Omnibus Test	QM	1.0958	0.9162	0.0004	1.7145	5.7933
		df	1	1	1	1	2
		Pvalue	0.2952	0.3385	0.9841	0.1904	0.0161
	Mixed-Effect Model	K	15	4	3	4	4
		τ2 estimator	REML	REML	REML	REML	REML
		τ2	0.0021 (SE = 0.0012)	0.0014 (SE = 0.0024)	0 (SE = 0.0082)	0.0030 (SE = 0.0037)	0 (SE = 0.0007)
		I2	72.32%	58.28%	0.00%	81.25%	0.00%
		H2	3.61	2.40	1.00	5.33	1.00
		R2	4.62%	10.55%	0.00%	27.48%	100.00%
	Model result	Zval	-1.0468	-0.9572	0.0200	-1.3094	-2.4069
		Pvalue	0.2952	0.3385	0.9841	0.1904	0.0161
	Residual Heterogeneity	QE	49.0157	0.0878	0.0147	11.4143	0.7443
		df	13	2	1	2	2
		Pvalue	< .0001	4.8661	0.9034	0.0033	0.6893

* All cohorts evaluated in different intervals were entered to the model.

† Data were sparce for outcome of TIA clinic cohort within 2 days

There were inadequate data for running a model for outcome of patients treated in TIA clinics within two days of index TIA. DL, DerSimonian and Laird. QM, indicates test statistic for the omnibus test of coefficients; df, degree of freedom; K, number of outcomes included in the model fitting; τ2, estimated amount of residual heterogeneity; I2 statistic, residual heterogeneity / unaccounted variability; H2, unaccounted variability / sampling variability; R2, amount of heterogeneity accounted.

Setting	Test	Parameter	Among all cohorts	Within 2 days	Within 7 days	Within 30 days	Within 90 days
Emergency Department	Omnibus Test	QM	9.6061	3.5343	7.2329	1.4271	1.8490
		df	2	1	1	2	1

© 2022 Shahjouei S et al. *JAMA Network Open.*

		**Pvalue**	0.0019	0.0601	0.0072	0.2322	0.1739
	**Mixed-Effect Model**	**K**	13	4	3	3	3
		**τ2 estimator**	REML	REML	REML	REML	REML
		**τ2**	0.0002 (SE = 0.0004)	0 (SE = 0.0011)	0.0001 (SE = 0.0014)	0 (SE = 0.0014)	0 (SE = 0.0014)
		**I2**	15.24%	0.00%	9.37%	0.00%	0.00%
		**H2**	1.18	1.00	1.10	1.00	1.00
		**R2**	76.65%	100.00%	97.38%	100.00%	100.00%
	**Model result**	**Zval**	3.0994	1.8800	2.6894	1.1946	1.3598
		**Pvalue**	0.0019	0.0601	0.0072	0.2322	0.1739
	**Residual Heterogeneity**	**QE**	10.4228	0.7267	1.1033	0.7898	0.4800
		**df**	11	2	1	1	1
		**Pvalue**	0.4928	0.6953	0.2935	0.0711	0.4884
**Unspecified Setting**	**Omnibus Test**	**QM**	0.6934	9.3576	2.1743	5.9495	0.0016
		**df**	1	2	1	1	1
		**Pvalue**	0.4050	0.0022	0.1403	0.0147	0.9686
	**Mixed-Effect Model**	**K**	22	4	2	3	9
		**τ2 estimator**	REML	REML	REML	REML	REML
		**τ2**	0.0058 (SE = 0.0020)	0.0000 (SE = 0.0003)	0.0042 (SE = 0.0033)	0 (SE = 0.0005)	0.0089 (SE = 0.0051)
		**I2**	94.34%	0.70%	91.78%	0.00%	95.21%
		**H2**	17.67	1.01	12.16	1.00	20.87
		**R2**	0.00%	99.86%	23.55%	100.00%	0.00%
	**Model result**	**Zval**	0.8327	3.0590	1.4746	2.4392	0.0394
		**Pvalue**	0.4050	0.0022	0.1403	0.0147	0.9686
	**Residual Heterogeneity**	**QE**	252.4444	1.9189	28.7424	0.0518	93.2917
		**df**	20	2	4	1	7
		**Pvalue**	< .0001	0.3831	< .0001	0.8200	< .0001

* All cohorts evaluated in different intervals were entered to the model.

There were inadequate data for running a model for outcome of patients treated in TIA clinics within two days of index TIA. DL, DerSimonian and Laird. QM, indicates test statistic for the omnibus test of coefficients; df, degree of freedom; K, number of outcomes included in the model fitting; τ2, estimated amount of residual heterogeneity; I2 statistic, residual heterogeneity / unaccounted variability; H2, unaccounted variability / sampling variability; R2, amount of heterogeneity accounted.

© 2022 Shahjouei S et al. *JAMA Network Open.*

eTable 3. Excluded Studies

Study	Reason for exclusion	Replaced study
**Akijian et al., 2017**[1]	Duplicate Data (North Dublin Stroke Study)	Sheehan et al., 2010[2]
**Amarenco et al., 2012**[3]	Duplicate Data (SOS-TIA)	Lavallée et al., 2017[4]
**Amarenco et al., 2016**[5]	No Outcome of Interest	
**Amarenco et al., 2018**[6]	No Outcome of Interest	
**Amort et al., 2011**[7]	Duplicate Data (Basel Stroke Unit Program)	Engelter et al., 2012[8]
**Arhami Dolatabadi et al., 2013**[9]	Evaluation Time Not Available	
**Arling et al., 2019**[10]	No Outcome of Interest	
**Baker et al., 1968**[11]	Evaluation Time Not Available	
**Banerjee et al., 2009**[12]	No Outcome of Interest	
**Barber et al., 2016**[13]	No Outcome of Interest	
**Barton et al., 2017**[14]	No Outcome of Interest	
**Benavente et al. 2013**[15]	No Outcome of Interest	
**Birns et al. 2006**[16]	No Outcome of Interest	
**Boden-Albala et al., 2019**[17]	No Outcome of Interest	
**Bose et al., 2017**[18]	Review Article	
**Bots et al., 1997**[19]	Evaluation Time Not Available	
**Boulanger et al., 2018**[20]	Guideline or Study Design Descriptions	
**Bradley et al., 2013**[21]	No Outcome of Interest	
**Brainin et al., 1995**[22]	Stroke Diagnosis Prior to Index Event	
**Bravata et al., 2018**[23]	No Outcome of Interest	
**Bravata et al., 2019**[24]	No Outcome of Interest	
**Burn et al., 1994**[25]	Duplicate Data (OCSP)	Johnston et al., 2007[26]
**Calvet et al., 2007**[27]	Duplicate Data	Calvet et al., 2009[28]
**Cameron et al., 2011**[29]	No Outcome of Interest	
**Candelise et al., 1986**[30]	Evaluation Time Not Available	
**Chandratheva et al., 2010**[31]	Duplicate Data (OXVASC)	Johnston et al., 2007[26]
**Chang et al., 2017**[32]	Review Article	
**Clissold et al., 2020**[33]	Review Article	
**Cosker et al., 2015**[34]	No Outcome of Interest	
**Couillard et al., 2009**[35]	Review Article	
**Coull et al., 2004**[36]	Duplicate Data (OXVASC)	Johnston et al., 2007[26]
**Cruz-Flores et al., 2017** [37]	Guideline or Study Design Descriptions	
**Dawson et al., 2008**[38]	No Outcome of Interest	
**Dennis et al., 1989**[39]	Review Article	
**Dennis et al., 1990**[40]	Duplicate Data (OCSP)	Johnston et al., 2007[26]
**Dennis et al., 1989**[39]	Duplicate Data (OCSP)	Johnston et al., 2007[26]
**Dolmans et al. 2019**[41]	Review Article	
**Dutta et al. 2016**[42]	No Outcome of Interest	
**Edlow et al., 2018**[43]	Guideline or Study Design Descriptions	
**Eliasziw et al., 1995**[44]	Evaluation Time Not Available	
**Evans et al., 1994**[45]	Evaluation Time Not Available	
**Falke et al., 1994**[46]	Evaluation Time Not Available	
**Feigin et al., 2000**[47]	No Outcome of Interest	
**Ferro et al. 1994**[48]	Outcome Reported as TIA or Stroke Recurrence	
**Fiorelli et al., 1995**[49]	No Outcome of Interest	
**Floßmann et al., 2003**[50]	Review Article	
**Fratiglioni et al., 1989**[51]	No Outcome of Interest	
**Fratiglioni et al., 1991**[52]	No Outcome of Interest	
**Friday et al., 1997**[53]	Evaluation Time Not Available	

© 2022 Shahjouei S et al. *JAMA Network Open.*

Study	Reason for exclusion	Replaced study
**Gennesseaux et al., [54]**	Guideline or Study Design Descriptions	
**Giles et al., 2006[55]**	Review Article	
**Giles et al., 2007[56]**	Review Article	
**Gilles et al. 2007[57]**	Study Cohort Includes Patients without Cerebral Ischemia	
**Gommans et al. 2009[58]**	Guideline or Study Design Descriptions	
**Graham et al. 2019[59]**	Study Cohort Includes Patients without Cerebral Ischemia	
**Griffith et al. 2014[60]**	Study Cohort Includes Patients without Cerebral Ischemia	
**Gulli et al., 2013[61]**	Evaluation Time Not Available	
**Hankey et al., 1991[62]**	Evaluation Time Not Available	
**Hankey et al., 1992[63]**	Evaluation Time Not Available	
**Hayashi et al. 2014[64]**	Evaluation Time Not Available	
**Hier et al., 1991[65]**	No Outcome of Interest	
**Holzer et al., 2010[66]**	Evaluation Time Not Available	
**Hörer et al., 2011[67]**	Outcome Reported as TIA or Stroke Recurrence	
**Hoshino et al., 2013[68]**	Stroke Diagnosis Prior to Index Event	
**Hoshino et al., 2013[69]**	Stroke Diagnosis Prior to Index Event	
**Hosier et al. 2016[70]**	No Outcome of Interest	
**Howard et al., 1987[71]**	Evaluation Time Not Available	
**Jeerakathil et al., 2014[72]**	Guideline or Study Design Descriptions	
**Johnson et al. 1986[73]**	Evaluation Time Not Available	
**Johnston et al., 2000[74]**	Duplicate Data (KMPCP)	Johnston et al., 2007[26]
**Joundi et al. 2017[75]**	Review Article	
**Kandiyali et al. 2017[76]**	Review Article	
**Karpal et al. 2016[77]**	Outcome Reported as TIA or Stroke Recurrence	
**Kernan et al., 2000[78]**	No Outcome of Interest	
**Kimura et al., 2004[79]**	Evaluation Time Not Available	
**Kokubo, 2014 [80]**	Review Article	
**Lai et al. 1990, [81]**	No Outcome of Interest	
**Lavallée et al., 2007[82]**	Duplicate Data(SOS-TIA)	Lavallée et al., 2017[4]
**Lee et al. 2015[83]**	No Outcome of Interest	
**Lesenskyj et al. 2016[84]**	No Outcome of Interest	
**Levi et al., 2019[85]**	Guideline or Study Design Descriptions	
**Loeb et al. 1978[86]**	Evaluation Time Not Available	
**Lovette et al. 2003[87]**	Duplicate Data(OCSP)	Johnston et al., 2007[26]
**Luengo-Fernandez et al. 2013[88]**	No Outcome of Interest	
**Madsen et al. 2019[89]**	Commentary	
**Magin et al. 2013[90]**	No Outcome of Interest	
**Martí-Vilalta et al., 1979[91]**	Evaluation Time Not Available	
**Mas et al., 1995[92]**	Evaluation Time Not Available	
**Mendelowitz et al., 1981[93]**	Outcome Reported as TIA or Stroke Recurrence	
**Merwick et al. 2011[94]**	Review Article	
**Mettinger et al., 1984[95]**	Evaluation Time Not Available	
**Miller et al., 1975[96]**	Evaluation Time Not Available	
**Mortel et al., 1996[97]**	Evaluation Time Not Available	
**Muuronen et al., 1982[98]**	Evaluation Time Not Available	
**Noris et al., 1991[99]**	Review Article	
**O'Brien et al., 2015[100]**	Evaluation Time Not Available	
**Ois et al., 2019[101]**	Evaluation Time Not Available	
**Ong et al. 2010[102]**	No Outcome of Interest	
**Park et al. 2017[103]**	No Outcome of Interest	
**Pendlebury et al., 2009[104]**	Review Article	

© 2022 Shahjouei S et al. *JAMA Network Open.*

Study	Reason for exclusion	Replaced study
**Purroy et al., 2012**[105]	Evaluation Time Not Available	
**Purroy et al., 2013**[106]	Duplicate Data (PROMAPA)	Purroy et al., 2014[107]
**Redgrave et al., 2007**[108]	Review Article	
**Rhoads et al., 1980** [109]	Evaluation Time Not Available	
**Rooji et al. 2012**[110]	Guideline or Study Design Descriptions	
**Rothwell et al., 2003**[111]	Review Article	
**Rothwell et al., 2005** [112]	Duplicate Data (OXVASC, OCSP)	Johnston et al., 2007[26]
**Rothwell et al., 2005**[113]	Duplicate Data (OXVASC, OCSP)	Johnston et al., 2007[26]
**Sales et al. 2015**[114]	No Outcome of Interest	
**Sanossian et al., 2008** [115]	Review Article	
**Shah et al., 2008**[116]	Review Article	
**Shah et al.,1995** [117]	No Outcome of Interest	
**Simonsen et al., 1981**[118]	Evaluation Time Not Available	
**Sobel et al., 1989**[119]	Evaluation Time Not Available	
**Stead et al., 2009**[120]	Duplicate Data (EDOU)	Stead et al., 2011[121]
**Streifler et al., 1995**[122]	Evaluation Time Not Available	
**Terént et al., 1980**[123]	Evaluation Time Not Available	
**Terént et al., 1989**[124]	Evaluation Time Not Available	
**Thacker et al., 2010**[125]	Stroke Diagnosis Prior to Index Event	
**Tomari et al. 2020**[126]	No Outcome of Interest	
**Toole et al., 1978**[127]	Evaluation Time Not Available	
**Torres Macho et al. 2011**[128]	Outcome Reported as TIA or Stroke Recurrence	
**Tsuda et al., 1983**[129]	Evaluation Time Not Available	
**Ueda et al., 1987**[130]	Evaluation Time Not Available	
**Valls et al., 2017**[131]	Review Article	
**Vandenbussche et al. 2017**[132]	Outcome Reported as TIA or Stroke Recurrence	
**Vilanova et al., 2015**[133]	Evaluation Time Not Available	
**Vrethem et al., 1990**[134]	Evaluation Time Not Available	
**Walker et al. 2012**[135]	No Outcome of Interest	
**Warrior et al. 2011**[136]	Review article	
**Weber et al. 2009**[137]	Review Article	
**Webster et al. 2011**[138]	No Outcome of Interest	
**Weimar et al., 2010**[139]	Evaluation Time Not Available	
**Weisberg et al., 1991**[140]	Evaluation Time Not Available	
**Whisnant et al., 1987**[141]	Evaluation Time Not Available	
**Widjaja et al. 2004**[142]	No Outcome of Interest	
**Wijk et al., 2005**[143]	Evaluation Time Not Available	
**Wu et al., 2007**[144]	Review Article	
**Zhong et al. 2016**[145]	Review Article	

© 2022 Shahjouei S et al. *JAMA Network Open.*

eTable 4. Heterogeneity and Risk of Stroke Assessment Based on Different Estimators

		Within 2 Days						Within 7 Days					
**Setting**	**Estimator**	**Q**	**P**	**I2**	**Risk**	**Lower CI**	**Upper CI**	**Q**	**P**	**I2**	**Risk**	**Lower CI**	**Upper CI**
**TIA Clinic**	**DL**	6.868	0.231	27.204	0.003	0.000	0.011	7.411	0.284	19.041	0.010	0.003	0.019
**TIA Clinic**	**HE**	6.868	0.231	0.000	0.004	0.001	0.009	7.411	0.284	0.000	0.011	0.005	0.017
**TIA Clinic**	**HS**	6.868	0.231	8.601	0.003	0.000	0.009	7.411	0.284	3.803	0.010	0.005	0.017
**TIA Clinic**	**ML**	6.868	0.231	22.436	0.003	0.000	0.010	7.411	0.284	16.479	0.010	0.003	0.018
**TIA Clinic**	**REML**	6.868	0.231	37.571	0.003	0.000	0.012	7.411	0.284	29.266	0.010	0.003	0.020
**TIA Clinic**	**SJ**	6.868	0.231	36.995	0.003	0.000	0.012	7.411	0.284	44.292	0.010	0.002	0.022
**TIA Clinic**	**EB**	6.868	0.231	17.697	0.005	0.001	0.009	7.411	0.284	11.569	0.011	0.005	0.020
**Inpatient**	**DL**	20.430	0.085	36.367	0.005	0.001	0.010	16.410	0.127	32.966	0.011	0.005	0.020
**Inpatient**	**HE**	20.430	0.085	0.000	0.004	0.001	0.007	16.410	0.127	0.000	0.010	0.005	0.016
**Inpatient**	**HS**	20.430	0.085	30.387	0.005	0.001	0.010	16.410	0.127	24.471	0.011	0.005	0.019
**Inpatient**	**ML**	20.430	0.085	37.020	0.005	0.001	0.010	16.410	0.127	36.406	0.012	0.004	0.021
**Inpatient**	**REML**	20.430	0.085	40.637	0.005	0.001	0.011	16.410	0.127	44.119	0.012	0.004	0.022
**Inpatient**	**SJ**	20.430	0.085	38.184	0.005	0.001	0.010	16.410	0.127	46.253	0.012	0.004	0.022
**Inpatient**	**EB**	20.430	0.085	23.593	0.005	0.001	0.009	16.410	0.127	30.550	0.011	0.005	0.020
**Emergency Department**	**DL**	6.390	0.495	0.000	0.019	0.013	0.026	15.919	0.044	49.745	0.034	0.023	0.047
**Emergency Department**	**HE**	6.390	0.495	0.000	0.019	0.013	0.026	15.919	0.044	18.572	0.034	0.025	0.044
**Emergency Department**	**HS**	6.390	0.495	0.000	0.019	0.013	0.026	15.919	0.044	42.138	0.034	0.024	0.046
**Emergency Department**	**ML**	6.390	0.495	0.000	0.019	0.013	0.026	15.919	0.044	41.539	0.034	0.024	0.046
**Emergency Department**	**REML**	6.390	0.495	7.018	0.019	0.012	0.027	15.919	0.044	48.384	0.034	0.023	0.047
**Emergency Department**	**SJ**	6.390	0.495	35.904	0.018	0.009	0.028	15.919	0.044	61.739	0.034	0.021	0.049
**Emergency Department**	**EB**	6.390	0.495	0.000	0.019	0.013	0.026	15.919	0.044	47.502	0.034	0.023	0.047
**Unspecified Settings**	**DL**	582.641	0.000	96.739	0.022	0.011	0.035	707.647	0.000	96.467	0.034	0.021	0.048
**Unspecified Settings**	**HE**	582.641	0.000	94.005	0.022	0.013	0.031	707.647	0.000	94.523	0.034	0.023	0.045
**Unspecified Settings**	**HS**	582.641	0.000	95.570	0.022	0.012	0.033	707.647	0.000	95.340	0.034	0.023	0.046
**Unspecified Settings**	**ML**	582.641	0.000	93.422	0.022	0.014	0.031	707.647	0.000	94.112	0.033	0.024	0.045
**Unspecified Settings**	**REML**	582.641	0.000	93.784	0.022	0.013	0.031	707.647	0.000	94.363	0.034	0.023	0.045
**Unspecified Settings**	**SJ**	582.641	0.000	93.991	0.022	0.013	0.031	707.647	0.000	94.530	0.034	0.023	0.045
**Unspecified Settings**	**EB**	582.641	0.000	93.828	0.022	0.013	0.031	707.647	0.000	94.434	0.034	0.023	0.045

DL, DerSimonian and Laird estimator; HS, Hunter and Schmidt estimator; Hedges (HE) ML, Maximum Likelihood estimator; REML, Restricted Maximum Likelihood estimator; SJ, Sidik and Jonkman estimator; EB, empirical Bayes, estimator; Q, Cochran's Q test ($\chi2$ test for heterogeneity); p, p-value for Q; I2, I2 statistic; Risk, risk of subsequent stroke following index event.

© 2022 Shahjouei S et al. *JAMA Network Open.*

		Within 30 Days							Within 90 Days					
**Setting**	**Estimator**	**Q**	**P**	**I2**	**Risk**	**Lower CI**	**Upper CI**		**Q**	**P**	**I2**	**Risk**	**Lower CI**	**Upper CI**
**TIA Clinic**	DL	11.699	0.069	48.715	0.014	0.005	0.026		17.317	0.138	30.705	0.021	0.015	0.028
**TIA Clinic**	HE	11.699	0.069	0.000	0.016	0.010	0.023		17.317	0.138	0.000	0.023	0.018	0.028
**TIA Clinic**	HS	11.699	0.069	34.036	0.014	0.006	0.024		17.317	0.138	23.464	0.021	0.016	0.027
**TIA Clinic**	ML	11.699	0.069	44.971	0.014	0.005	0.025		17.317	0.138	34.516	0.021	0.015	0.028
**TIA Clinic**	REML	11.699	0.069	52.824	0.013	0.004	0.026		17.317	0.138	40.560	0.021	0.014	0.028
**TIA Clinic**	SJ	11.699	0.069	44.334	0.014	0.005	0.025		17.317	0.138	43.473	0.021	0.014	0.028
**TIA Clinic**	EB	11.699	0.069	36.133	0.016	0.006	0.030		17.317	0.138	25.078	0.028	0.021	0.035
**Inpatient**	**DL**	60.791	0.000	78.615	0.016	0.007	0.027		47.407	0.003	49.375	0.027	0.021	0.035
**Inpatient**	**HE**	60.791	0.000	75.010	0.016	0.007	0.026		47.407	0.003	0.000	0.026	0.022	0.030
**Inpatient**	**HS**	60.791	0.000	11.759	0.011	0.008	0.015		47.407	0.003	45.887	0.027	0.021	0.034
**Inpatient**	**ML**	60.791	0.000	86.053	0.016	0.006	0.030		47.407	0.003	51.626	0.028	0.021	0.035
**Inpatient**	**REML**	60.791	0.000	87.280	0.016	0.006	0.031		47.407	0.003	54.495	0.028	0.021	0.035
**Inpatient**	**SJ**	60.791	0.000	86.316	0.016	0.006	0.031		47.407	0.003	65.554	0.028	0.020	0.037
**Inpatient**	**EB**	60.791	0.000	85.419	0.016	0.006	0.030		47.407	0.003	52.756	0.028	0.021	0.035
**Emergency Department**	DL	22.936	0.001	73.840	0.036	0.017	0.061		4.337	0.631	0.000	0.035	0.025	0.045
**Emergency Department**	HE	22.936	0.001	74.317	0.036	0.016	0.061		4.337	0.631	0.000	0.035	0.025	0.045
**Emergency Department**	HS	22.936	0.001	67.302	0.036	0.019	0.059		4.337	0.631	0.000	0.035	0.025	0.045
**Emergency Department**	ML	22.936	0.001	73.451	0.036	0.017	0.061		4.337	0.631	0.000	0.035	0.025	0.045
**Emergency Department**	REML	22.936	0.001	78.470	0.035	0.015	0.063		4.337	0.631	0.000	0.035	0.025	0.045
**Emergency Department**	SJ	22.936	0.001	80.112	0.035	0.014	0.064		4.337	0.631	40.154	0.033	0.020	0.048
**Emergency Department**	EB	22.936	0.001	78.469	0.035	0.015	0.063		4.337	0.631	0.000	0.035	0.025	0.045
**Unspecified Settings**	DL	240.300	0.000	92.926	0.042	0.029	0.058		1230.686	0.000	97.562	0.060	0.047	0.075
**Unspecified Settings**	HE	240.300	0.000	94.114	0.042	0.028	0.059		1230.686	0.000	98.343	0.060	0.044	0.078
**Unspecified Settings**	HS	240.300	0.000	91.884	0.042	0.030	0.056		1230.686	0.000	96.225	0.060	0.049	0.072
**Unspecified Settings**	ML	240.300	0.000	93.329	0.042	0.029	0.058		1230.686	0.000	98.250	0.060	0.045	0.077
**Unspecified Settings**	REML	240.300	0.000	93.753	0.042	0.028	0.059		1230.686	0.000	98.310	0.060	0.045	0.078
**Unspecified Settings**	SJ	240.300	0.000	94.047	0.042	0.028	0.059		1230.686	0.000	98.332	0.060	0.045	0.078
**Unspecified Settings**	EB	240.300	0.000	93.933	0.042	0.028	0.059		1230.686	0.000	98.325	0.060	0.045	0.078

DL, DerSimonian and Laird estimator; HS, Hunter and Schmidt estimator; Hedges (HE) ML, Maximum Likelihood estimator; REML, Restricted Maximum Likelihood estimator; SJ, Sidik and Jonkman estimator; EB, empirical Bayes, estimator; Q, Cochran's Q test ($\chi 2$ test for heterogeneity); p, p-value for Q; I2, I2 statistic; Risk, risk of subsequent stroke following index event.

© 2022 Shahjouei S et al. *JAMA Network Open.*

eTable 5. Comparison of Risk Estimates

Comparison	Day Two			Day Seven			Day Thirty			Day Ninety		
**Meta-Analysis of All Cohorts**	**QE**	**QEP**	**I^2**	**QE**	**QEP**	**I^2**	**QE**	**QEP**	**I^2**	**QE**	**QEP**	**I^2**
**TIA Clinic versus Inpatient**	0.14	0.71	0.00	0.09	0.76	0.00	0.12	0.73	0.00	1.94	0.16	48.49
TIA Clinic versus Emergency Department	7.26	0.01	86.22	9.72	0.00	89.71	3.05	0.08	67.24	5.40	0.02	81.49
TIA Clinic versus Unspecified Setting	8.74	0.00	88.56	10.21	0.00	90.20	8.93	0.00	88.81	23.42	0.00	95.73
Inpatient versus Emergency Department	9.42	0.00	89.39	8.22	0.00	87.83	2.04	0.15	51.07	1.23	0.27	18.74
Inpatient versus Unspecified Setting	11.20	0.00	91.07	8.64	0.00	88.43	6.21	0.01	83.88	14.62	0.00	93.16
Emergency Department versus Unspecified Setting	0.24	0.62	0.00	0.00	0.96	0.00	0.21	0.65	0.00	7.13	0.01	85.97
**Sensitivity Meta-Analysis of Prospective Cohorts**	**QE**	**QEP**	**I^2**	**QE**	**QEP**	**I^2**	**QE**	**QEP**	**I^2**	**QE**	**QEP**	**I^2**
**TIA Clinic versus Inpatient**	0.01	0.94	0.00	0.06	0.81	0.00	0.00	1.00	0.00	0.55	0.46	0.00
TIA Clinic versus Emergency Department	2.28	0.13	56.19	7.61	0.01	86.86	1.52	0.22	34.03	2.70	0.10	62.96
TIA Clinic versus Unspecified Setting	5.88	0.02	83.01	7.28	0.01	86.26	3.98	0.05	74.90	10.85	0.00	90.78
Inpatient versus Emergency Department	2.71	0.10	63.13	12.62	0.00	92.07	1.46	0.23	31.67	1.26	0.26	20.66
Inpatient versus Unspecified Setting	9.01	0.00	88.90	13.78	0.00	92.74	3.38	0.07	70.44	8.31	0.00	87.96
Emergency Department versus Unspecified Setting	0.17	0.68	0.00	0.15	0.70	0.00	0.10	0.75	0.00	2.42	0.12	58.67

Comparisons were conducted under restricted maximum likelihood (REML) estimator. Sensitivity Analysis included the prospective cohorts recruited after 2000. QE indicates test statistics for the tests of heterogeneity; QEP, p-values for the tests of heterogeneity; I2, I2 statistic.

© 2022 Shahjouei S et al. *JAMA Network Open.*

eTable 6. Publication Bias Assessment

Interval	Setting	Egger Bias Test	Begg-Mazumdar Rank Correlation
Within 2 Days	TIA Clinic	z = 0.470, p = 0.638	Kendall's τ = -0.200, p = 0.719
	Inpatient	z = 0.768, p = 0.442	Kendall's τ = -0.033, p = 0.914
	Emergency Room	z = -1.408, p = 0.159	Kendall's τ = -0.357, p = 0.275
	Unspecified Settings	z = 1.088, p = 0.277	Kendall's τ = -0.116, p = 0.501
Within 7 Days	TIA Clinic	z = 0.773, p = 0.440	Kendall's τ = 0.143, p = 0.773
	Inpatient	z = 0.192, p = 0.848	Kendall's τ = 0.000, p = 1.000
	Emergency Room	z = -1.050, p = 0.294	Kendall's τ = -0.167, p = 0.612
	Unspecified Settings	z = 1.449, p = 0.148	Kendall's τ = -0.052, p = 0.727
Within 30 Days	TIA Clinic	z = -0.152, p = 0.879	Kendall's τ = -0.143, p = 0.773
	Inpatient	z = 0.355, p = 0.723	Kendall's τ = -0.055, p = 0.830
	Emergency Room	z = -0.883, p = 0.378	Kendall's τ = 0.047, p = 1.000
	Unspecified Settings	z = 0.857, p = 0.392	Kendall's τ = 0.164, p = 0.370
Within 90 Days	TIA Clinic	z = -1.6887, p = 0.091	Kendall's τ = -0.128, p = 0.590
	Inpatient	z = 0.0990, p = 0.921	Kendall's τ = 0.070, p = 0.624
	Emergency Room	z = -1.4499, p = 0.147	Kendall's τ = -0.619, p = 0.069
	Unspecified Settings	z = 1.0821, p = 0.279	Kendall's τ = 0.015, p = 0.920

© 2022 Shahjouei S et al. *JAMA Network Open.*

eTable 7. Risk-of-Bias Assessment Based on ROBINS-E Tool

Study	Confounding	Participants	Classification of Intervention	Deviation from Intended Intervention	Missing Data	Measurement of Outcome	Report of Result	Overall
Ahmad et al.,[146] 2013	Low	Low	Low	Low	No information	Low	Low	Moderate
Al-Khaled et al.,[147] 2013	Low	Low	Low	Low	Low	Low	Low	Low
Al-Khaled et al.,[148] 2014	Low	Low	No information	Low	Low	Low	Low	No information
Appelros et al.,[149] 2017	Moderate	Moderate	No information	Low	Low	Low	Low	No information
Arsava et al.,[150] 2011	Low	Low	Low	Low	Low	Low	Low	Low
Ay et al.,[151] 2009	Low	Low	Low	Low	Low	Low	Low	Low
Bonifati et al.,[152] 2011	Low	Low	Low	Low	Low	Low	Low	Low
Bos et al.,[153] 2007	Low	Low	No information	Low	Low	Low	Low	No information
Calvet et al.,[28] 2009	Low	Low	Low	Low	Low	Low	Low	Low
Cancelli et al.,[154] 2011	Low	Low	No information	Low	Low	Low	Low	No information
Chang et al.,[155] 2019	Low	Low	Low	Low	Low	Low	Low	Low
Cheong et al.,[156] 2019	Low	low	Low	Moderate	Low	Low	Moderate	Moderate
Correia et al.,[157] 2015	Low	Low	Low	Low	Low	Low	Low	Low
Coutts et al.,[158] 2008	Low	Low	Low	Low	Low	Low	Low	Low
Cucchiara et al.,[159] 2009	Low	Low	Low	Low	Low	Low	Low	Low
Dahlquist et al.,[160] 2019	Low	Low	Low	Low	Low	Low	Low	Low
De Marchis et al.,[161] 2014	Low	Low	Low	Low	Low	Low	Low	Low
Delgado et al.,[162] 2011	Low	Low	Low	Low	Low	Low	Low	Low
Dutta et al.,[42] 2016	Low	Low	Low	Low	Low	Low	Low	Low
Eliasziw et al.,[163] 2004	Low	Low	No information	Low	Low	Low	Low	No information
Engelter et al.,[164] 2012	Low	Low	Low	Moderate	Moderate	Moderate	Low	Moderate
Felgueiras et al.,[165] 2019	Low	Low	No information	Low	Low	Low	Low	No information

© 2022 Shahjouei S et al. *JAMA Network Open.*

**Fujinami et al.,[166] 2014**	Low	Low	No information	Low	Low	Low	Low	No informati on
**Geil et al.,[167] 2008**	Low	Low	Low	Low	Low	Low	Low	Low
**Ghia et al.,[168] 2011**	Low	Low	Low	Low	Low	Low	Low	Low
**Gladstone et al.,[169] 2004**	Low	Low	No information	Low	Low	Low	Low	No informati on
**Gon et al.,[170] 2015**	Low	Low	Low	Low	Low	Low	Low	Low
**Guarino et al.,[171] 2015**	Low	Low	Low	Low	Low	Low	Low	Low
**Harrison et al.,[172] 2010**	Low	Low	Low	No informati on	No informati on	Low	Low	No informati on
**Hill et al.,[173] 2004**	Low	Low	No information	Low	Low	Low	Low	No informati on
**Ildstat et al.,[174] 2019**	Low	Low	No information	Low	Low	Low	Low	No informati on
**Johnston et al.,[175] 2007**	Low	Low	No information	Low	Low	Low	Low	No informati on
**Jove et al.,[176] 2014**	Low	Low	Low	No informati on	Low	Low	Low	Moderate

© 2022 Shahjouei S et al. *JAMA Network Open.*

Study	Confounding	Participants	Classification of Intervention	Deviation from Intended Intervention	Missing Data	Measurement of Outcome	Report of Result	Overall
**Kiyohara et al.,[177] 2014**	Low	Low	No information	Low	Low	Low	Low	No information
**Kleindorfer et al.,[178] 2005**	Low	Low	No information	Low	Low	Moderate	Low	No information
**Lavallée et al.,[4] 2017**	Low	Low	Low	Low	Low	Low	Low	Low
**Lichtman et al.,[179] 2009**	Moderate	Low	Low	Low	Low	Low	Low	Moderate
**Lim et al.,[180] 2015**	Low	Low	No information	Low	Low	Low	Low	No information
**Lisabeth et al.,[181] 2004**	Low	Low	No information	Low	Low	Low	Low	No information
**Majidi et al.,[182] 2017**	Low	Low	Low	Low	Low	Low	Low	Low
**Montassier et al.,[183] 2013**	Low	Low	Low	Low	Low	Low	Low	Low
**Mowla et al., 2021**	Low	Low	Low	Low	Low	Low	Low	Low
**Nahab et al.,[184] 2012**	Low	Low	Low	Low	Low	Low	Low	Low
**Nguyen et al.,[185] 2010**	Low	Low	Low	Low	Low	Low	Low	Low
**Ohara et al.,[186] 2015**	Low	Low	No information	Low	Low	Low	Low	No information
**Ohara et al.,[187] 2019**	Moderate	Low	No information	Low	Low	Low	Low	No information
**Olivot et al.,[188] 2011**	Low	Low	low/ No information*	Low	Low	Low	Low	low/ No information*
**Ottaviani et al.,[189] 2016**	Low	Low	Low	Low	Low	Low	Low	Low
**Ovbiagele et al.,[190] 2008**	Low	Low	No information	Low	Low	Low	Low	No information
**Palomeras Soler et al.,[191] 2015**	Low	Low	Low	Low	Low	Low	Low	Low
**Perry et al.,[192] 2014**	Low	Moderate	No information	Low	Low	Low	Low	No information
**Purroy et al.,[193] 2014**	Low	Low	No information	Low	Low	Low	Low	No information
**Raposo et al.,[194] 2018**	Low	Low	Low	Low	Low	Low	Low	Low
**Raser et al.,[195] 2012**	Low	Low	Low	Low	Low	Low	Low	Low

© 2022 Shahjouei S et al. *JAMA Network Open.*

**Ricci et al.,[196] 1991**	Low	Low	No information	Low	Low	Low	Low	No information
**Ross et al.,[197] 2007**	Low	Low	Low	Low	Low	Low	Low	Low
**Rothwell et al.,[198] 2007**	Low	Low	No information	Low	Low	Low	Low	No information
**Sanders et al.,[199] 2012**	Low	Low	Low	Low	Low	Low	Low	Low
**Sciolla et al.,[200] 2008**	Low	Low	No information	Low	Low	Low	Low	No information
**Selvarajah et al.,[201] 2008**	Low	Low	No information	Low	Low	Low	Low	No information
**Sheehan et al.,[2] 2010**	Low	Low	No information	Low	Low	Low	Low	No information
**Song et al.,[202] 2013**	Low	Low	No information	Low	Low	Low	Low	No information
**Stead et al.,[120] 2009**	Low	Low	Low	Low	Low	Low	Low	Low
**Stead et al.,[203] 2011**	Low	Low	Low	Low	Low	Low	Low	Low
**Sundararajan et al.,[204] 2014**	Low	Low	No information	Low	Low	Low	Low	No information
**Tsivgoulis et al.,[205] 2010**	Low	Low	No information	Low	Low	Low	Low	No information

© 2022 Shahjouei S et al. *JAMA Network Open.*

Study		Confounding	Participants	Classification of Intervention	Deviation from Intended Intervention	Missing Data	Measurement of Outcome	Report of Result	Overall
**Vigen et al.,[206] 2018**		Low	Low	Low	Low	Low	Low	Low	Low
**Vora et al.,[207] 2015**		Low	Low	low/ No information	Low	Low	Low	Low	low/ No information*
**Wasserman et al.,[208] 2010**		Low	Low	Low	Low	Low	Low	Low	Low
**Weimar et al.,[209] 2009**		Low	Moderate	No information	Low	Low	Low	Low	No information
**Weitzel-Mudersbach et al.,[210] 2011**		Low	Low	Low/ No information*	Low	Low	Low	Low	low/ No information*
**Wu et al.,[211] 2009**		Low	Low	Low	Low	Low	Low	Low	Low
**Risk of bias by considering the distinct cohorts in each study**	**Low**	**98 (97.0%)**	**98 (97.0%)**	**64 (63.30%)**	**96 (95.0%)**	**98 (97.0%)**	**99 (98.0%)**	**99 (98.0%)**	**58 (57.4%)†**
	**Moderate**	**3 (3.0%)**	**3 (3.0%)**	**0 (0.0%)**	**3 (3.0%)**	**1 (1.0%)**	**2 (2.0%)**	**2 (2.0%)**	**6 (5.9%)**
	**High**	**0 (0.0%)**	**0 (0.0%)**	**0 (0.0%)**	**0 (0.0%)**	**0 (0.0%)**	**0 (0.0%)**	**0 (0.0%)**	**0 (0.0%)**
	**No Information**	**0 (0.0%)**	**0 (0.0%)**	**37 (36.6%)**	**2 (2.0%)**	**2 (2.0%)**	**0 (0.0%)**	**0 (0.0%)**	**37 (36.6%)**

* Some cohorts in this study had low risk of bias while there was no information regarding other cohort(s).

† This value is represented for all included cohorts in the study (110 cohorts). Among the 64 cohorts with specified setting (i.e. TIA clinic, inpatient, and ED) 58 (90.6%) cohorts had low and 6 (9.4%) cohorts had moderate overall risk of bias.

© 2022 Shahjouei S et al. *JAMA Network Open.*

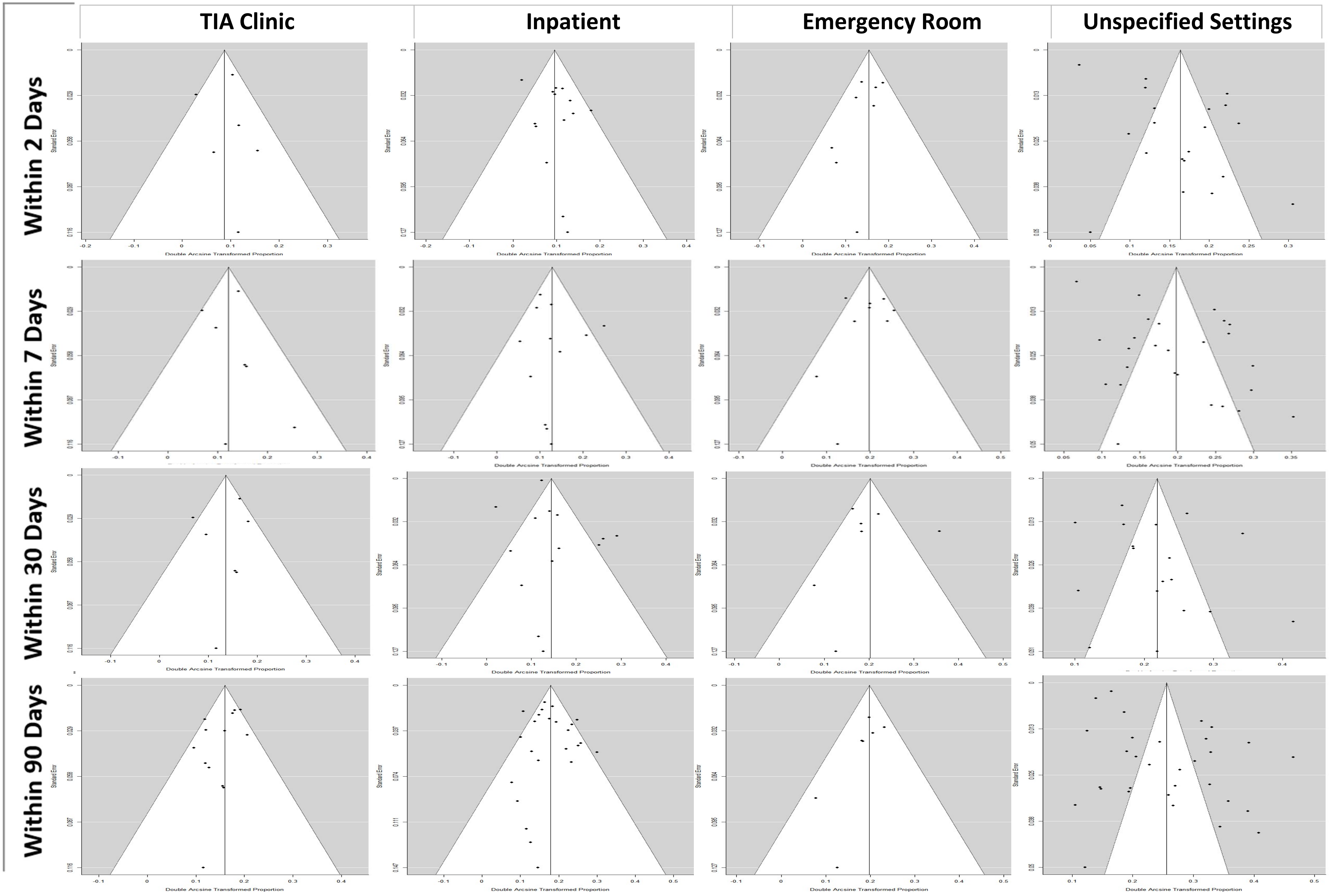

eFigure 1. Funnel Plots

©

eFigure 2. Risk of Subsequent Ischemic Stroke Within 2 Days

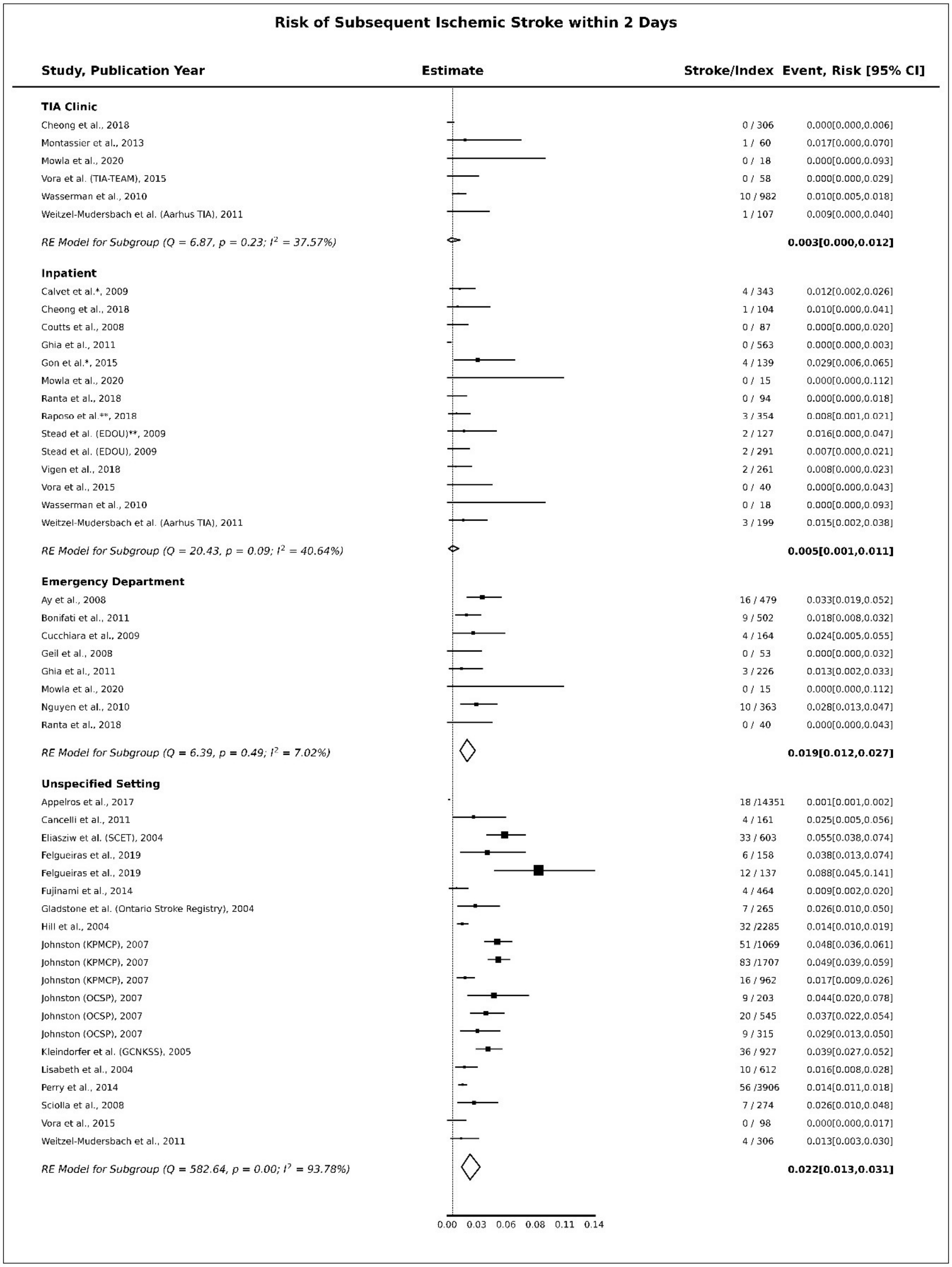

The risk estimate for inpatients was considered as the reference line. * indicates the Stroke Unit, ** indicates the Observation Unit.

© 2022 Shahjouei S et al. *JAMA Network Open.*

eFigure 3. Risk of Subsequent Ischemic Stroke Within 30 Days

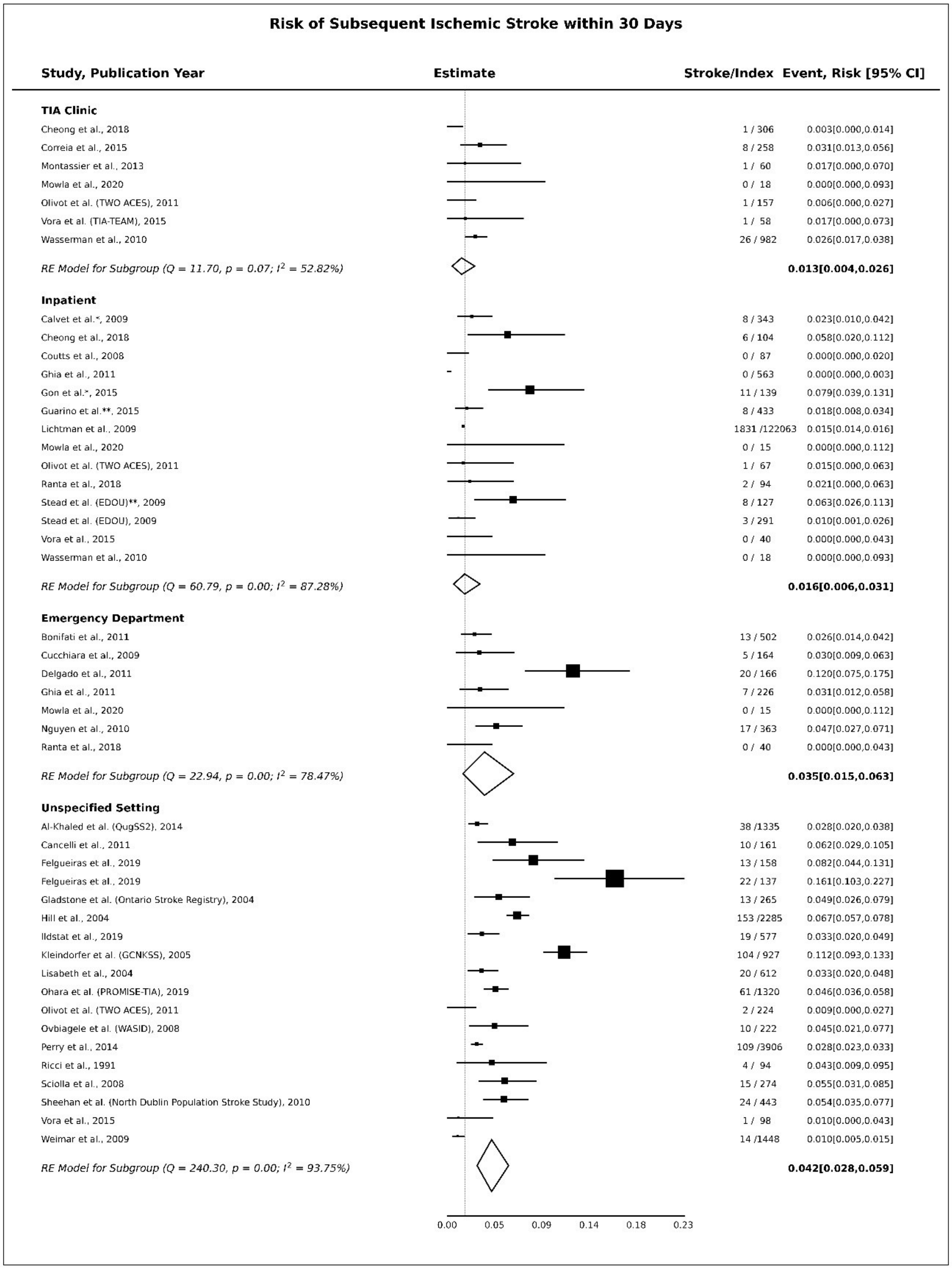

The risk estimate for inpatients was considered as the reference line. * indicates the Stroke Unit, ** indicates the Observation Unit.

© 2022 Shahjouei S et al. *JAMA Network Open.*

eFigure 4. Sensitivity Analysis: Risk of Subsequent Ischemic Stroke Within 2 Days

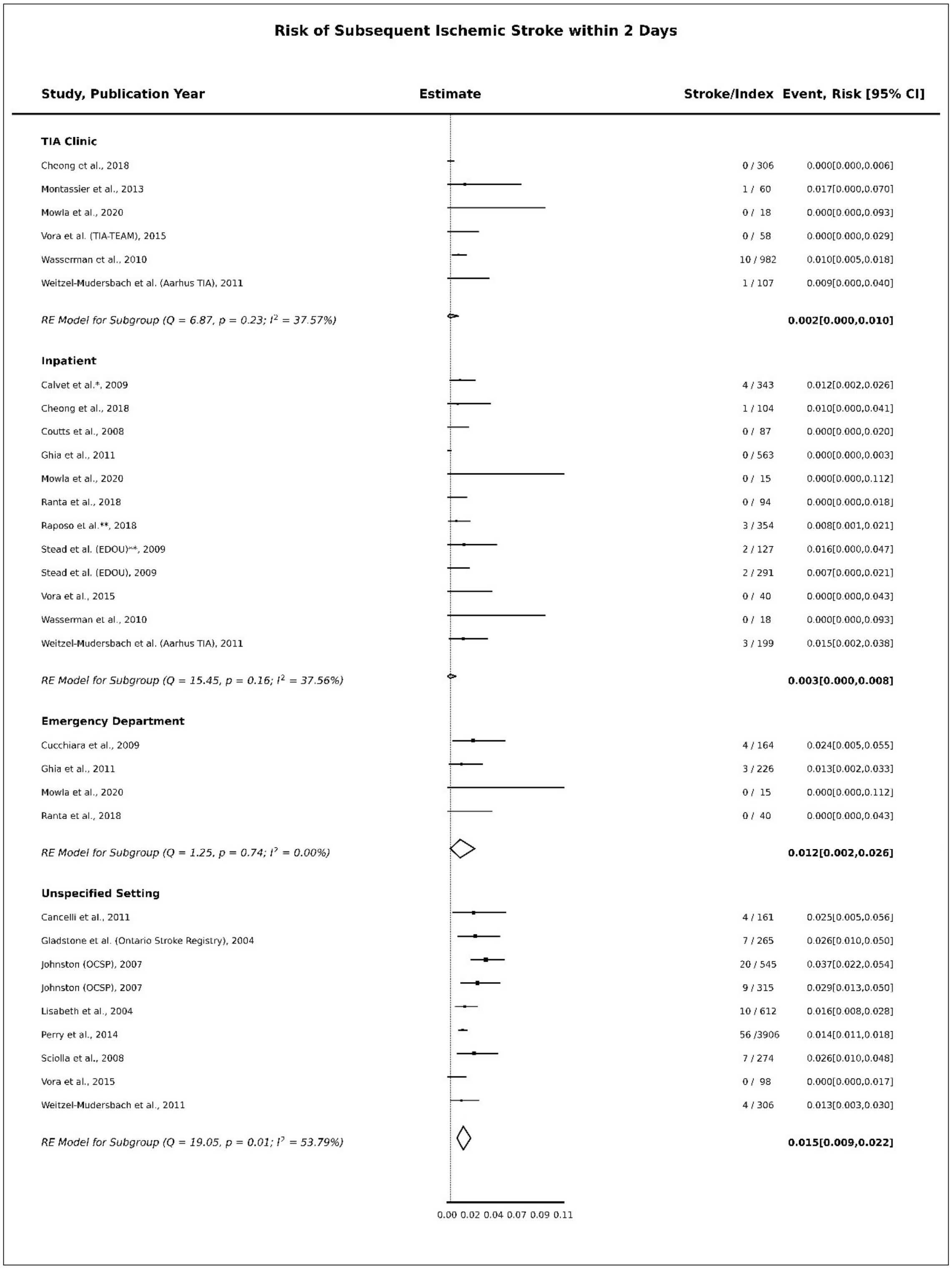

Sensitivity analysis included the prospective cohort of patients recruited after 2000.

The risk estimate for inpatients was considered as the reference line. * indicates the Stroke Unit, ** indicates the Observation Unit.

© 2022 Shahjouei S et al. *JAMA Network Open.*

eFigure 5. Sensitivity Analysis: Risk of Subsequent Ischemic Stroke Within 7 Days

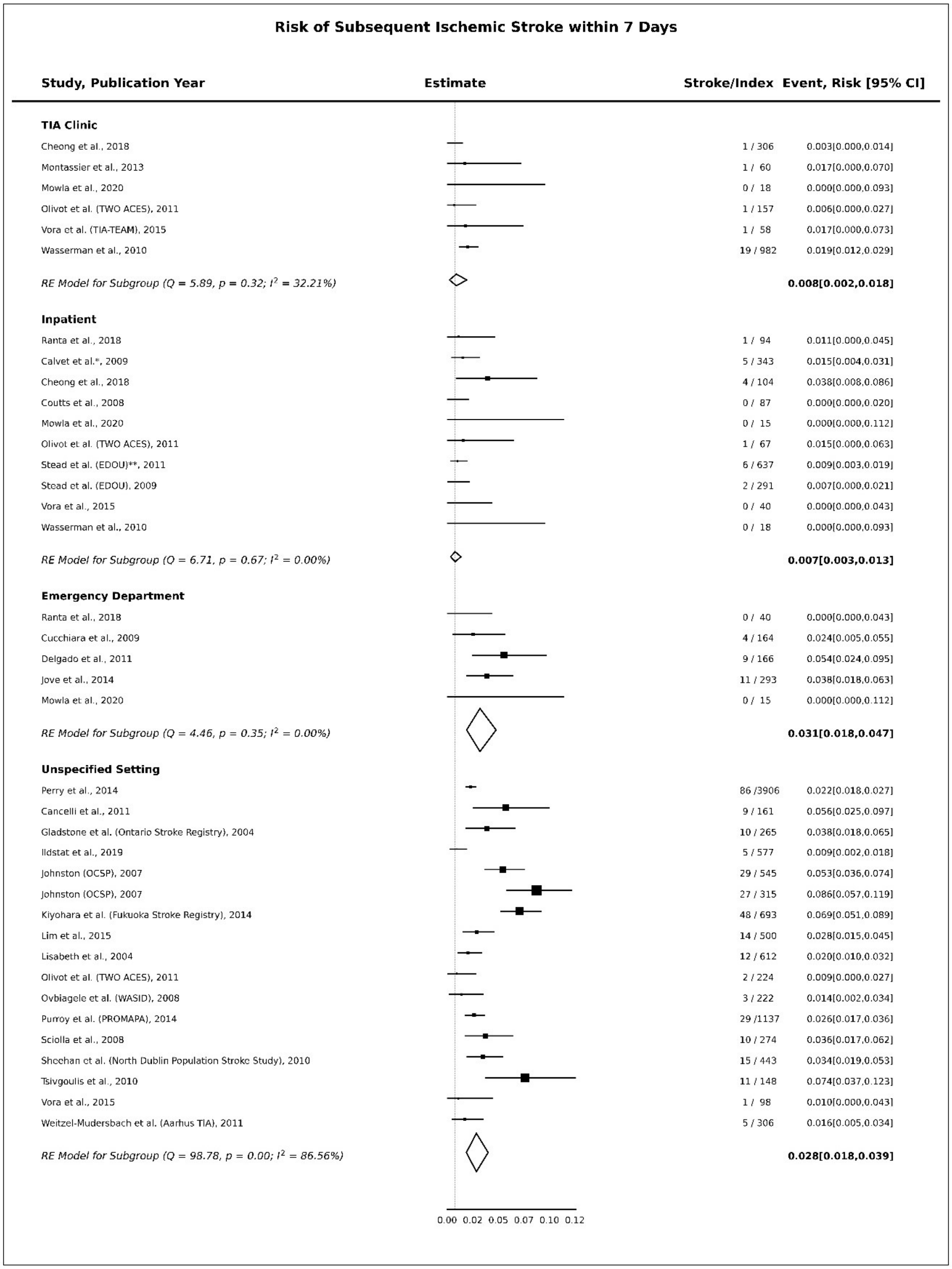

Sensitivity analysis included the prospective cohort of patients recruited after 2000.

The risk estimate for inpatients was considered as the reference line. * indicates the Stroke Unit, ** indicates the Observation Unit.

© 2022 Shahjouei S et al. *JAMA Network Open.*

eFigure 6. Sensitivity Analysis: Risk of Subsequent Ischemic Stroke Within 30 Days

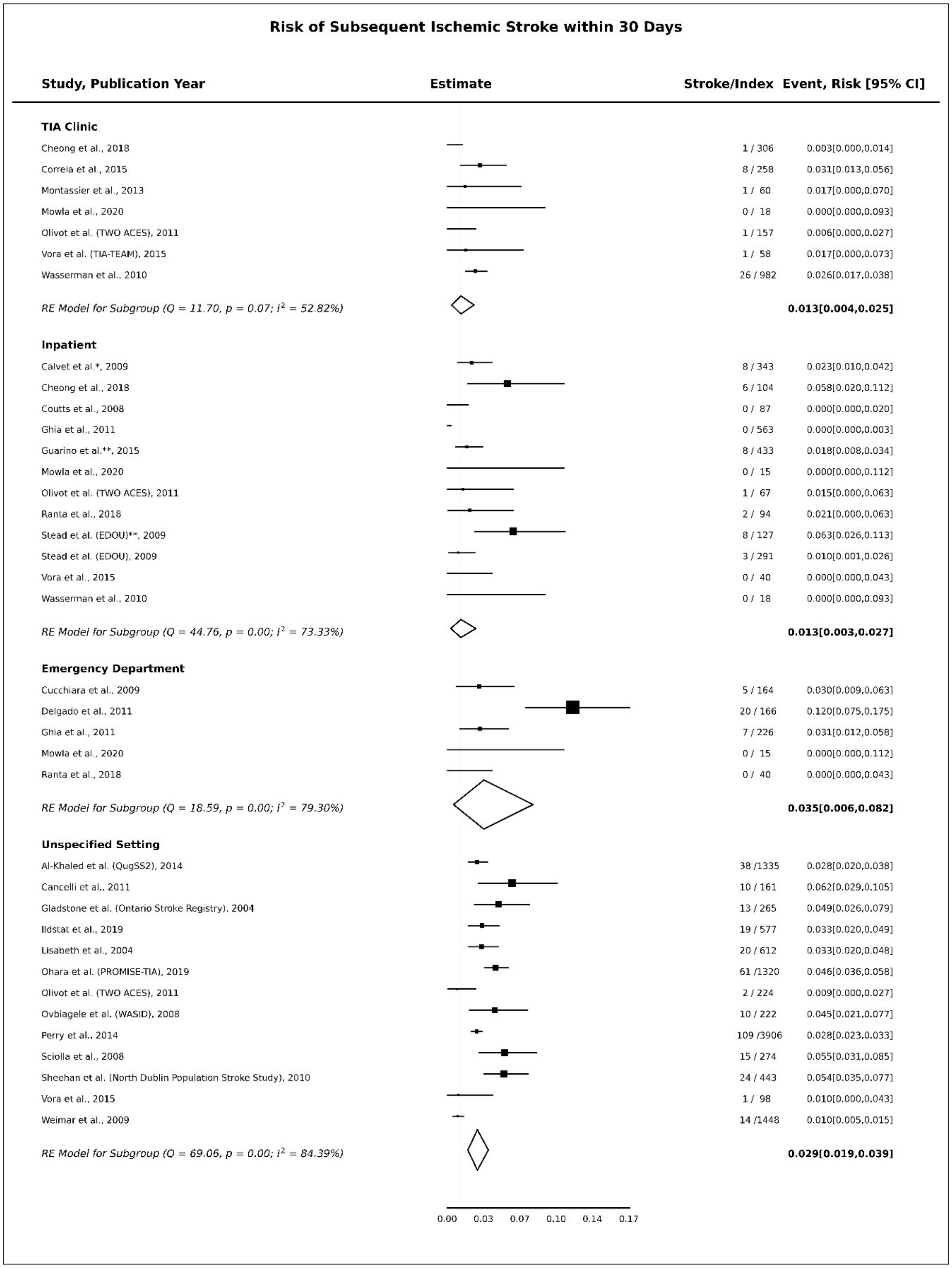

Sensitivity analysis included the prospective cohort of patients recruited after 2000.

The risk estimate for inpatients was considered as the reference line. * indicates the Stroke Unit, ** indicates the Observation Unit.

© 2022 Shahjouei S et al. *JAMA Network Open.*

eFigure 7. Sensitivity Analysis: Risk of Subsequent Ischemic Stroke Within 90 Days

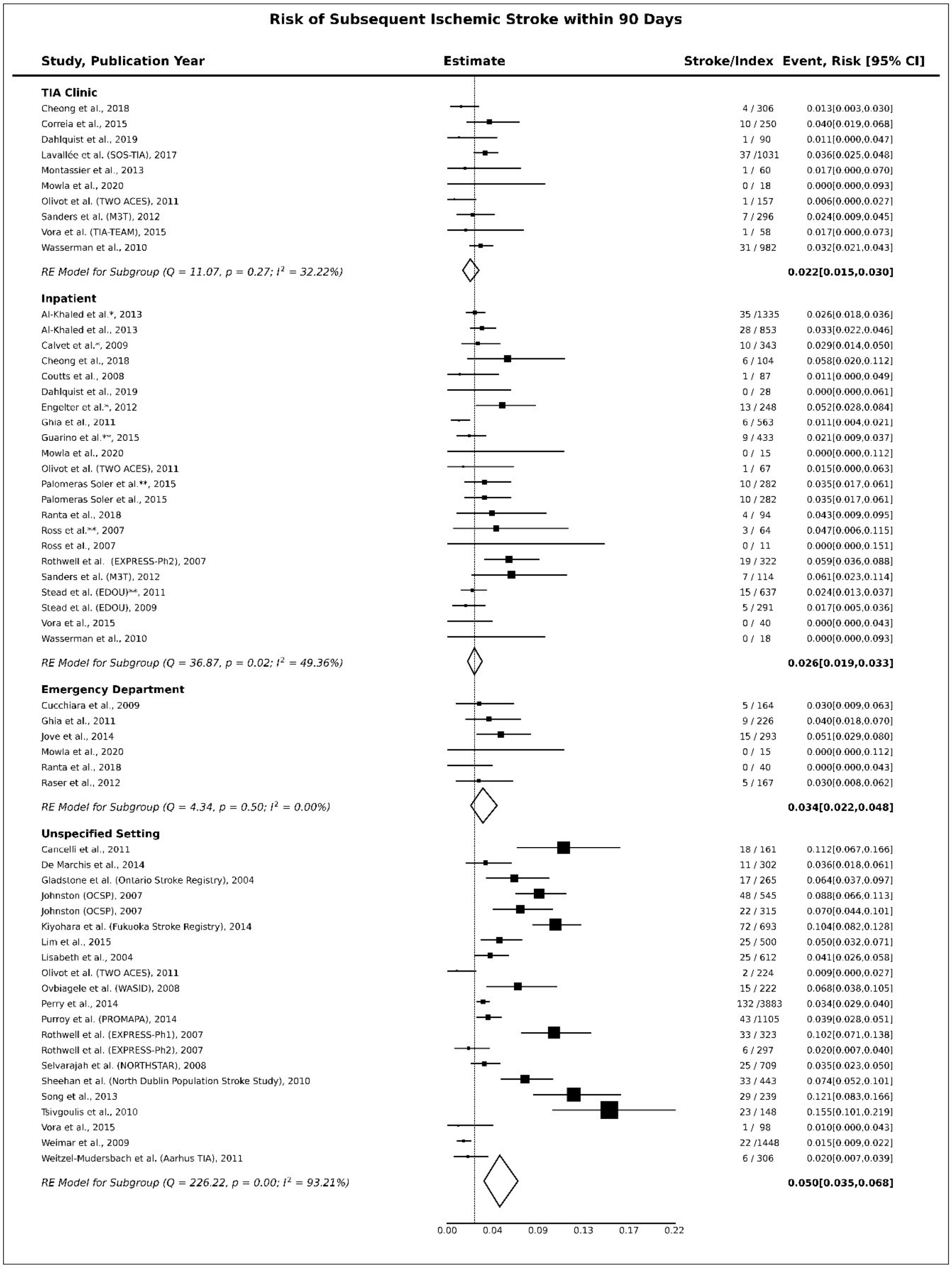

Sensitivity analysis included the prospective cohort of patients recruited after 2000.

The risk estimate for inpatients was considered as the reference line. * indicates the Stroke Unit, ** indicates the Observation Unit.

© 2022 Shahjouei S et al. *JAMA Network Open.*

eReferences.

1. Akijian L, Ní Chróinín D, Callaly E, Hannon N, Marnane M, Merwick Á, Sheehan Ó, Hayden D, Horgan G, Duggan J, et al. Why do transient ischemic attack patients have higher early stroke recurrence risk than those with ischemic stroke? Influence of patient behavior and other risk factors in the North Dublin Population Stroke Study. *Int. J. Stroke*. 2017;12:96–104.

2. Sheehan OC, Kyne L, Kelly LA, Hannon N, Marnane M, Merwick A, McCormack PME, Duggan J, Moore A, Moroney J, et al. Population-based study of ABCD2 score, carotid stenosis, and atrial fibrillation for early stroke prediction after transient ischemic attack: The North Dublin TIA study. *Stroke*. 2010;41:844–850.

3. Amarenco P, Labreuche J, Lavallée PC, PC L, Amarenco P, Labreuche J, Lavallée PC. Patients with transient ischemic attack with ABCD2 <4 can have similar 90-day stroke risk as patients with transient ischemic attack with ABCD2 ≥4. *Stroke (00392499)* [Internet]. 2012;43:863–865. Available from: https://ezproxy.uthsc.edu/login?url=http://search.ebscohost.com/login.aspx?direct=true&db=ccm&AN=108160245&site=ehost-live

4. Lavallée PC, Sissani L, Labreuche J, Meseguer E, Cabrejo L, Guidoux CC, Klein IF, Touboul P-JJ, Amarenco P, Lavallee PC, et al. Clinical significance of isolated atypical transient symptoms in a cohort with transient ischemic attack. *Stroke*. 2017;48:1495–1500.

5. Amarenco P, Lavallée PC, Labreuche J, Albers GW, Bornstein NM, Canhão P, Caplan LR, Donnan GA, Ferro JM, Hennerici MG, et al. One-year risk of stroke after transient ischemic attack or minor stroke. *N. Engl. J. Med.* 2016;374:1533–42.

6. Amarenco P, Lavallée PC, Monteiro Tavares L, Labreuche J, Albers GW, Abboud H, Anticoli S, Audebert H, Bornstein NM, Caplan LR, et al. Five-year risk of stroke after TIA or minor ischemic stroke. *N. Engl. J. Med.* 2018;378:2182–2190.

7. Amort M, Fluri F, Schäfer J, Weisskopf F, Katan M, Burow A, Bucher HC, Bonati LH, Lyrer PA, Engelter ST. Transient ischemic attack versus transient ischemic attack mimics: Frequency, clinical characteristics and outcome. *Cerebrovasc. Dis.* 2011;32:57–64.

8. Engelter ST, Amort M, Jax F, Weisskopf F, Katan M, Burow A, Bonati LH, Hatz F, Wetzel SG, Fluri F, et al. Optimizing the risk estimation after a transient ischaemic attack - The ABCDE⊕ score. *Eur. J. Neurol.* 2012;19:55–61.

9. Arhami Dolatabadi A, Meisami A, Hatamabadi H, Mansori B, Shahrami A, Amini A, Jamali K. Improving the prediction of stroke or death after transient ischemic attack (TIA) by adding diffusion-weighted imaging lesions and TIA etiology to the ABCD2 score. *J. Stroke Cerebrovasc. Dis.* 2013;22:e25–e30.

10. Arling G, Sico JJ, Reeves MJ, Myers L, Baye F, Bravata DM. Modelling care quality for patients after a transient ischaemic attack within the US Veterans Health Administration. *BMJ Open Qual.* 2019;8:e000641.

11. Baker RN, Ramseyer JC, Schwartz WS. Prognosis in patients with transient cerebral ischemic attacks. *Neurology*. 1968;18:1157–1165.

12. Banerjee S, Natarajan I, Biram R, Sutton K, Ekeng G, Ames D, Chataway J. FAST-TIA: A prospective evaluation of a nurse-led anterior circulation TIA clinic. *Postgrad. Med. J.* 2009;85:637–642.

13. Barber PA, Krishnamurthi R, Parag V, Anderson NE, Ranta A, Kilfoyle D, Wong E, Green G, Arroll B, Bennett DA, et al. Incidence of Transient Ischemic Attack in Auckland, New Zealand, in 2011 to 2012. Stroke. 2016;47:2183–2188.

14. Barton P, Sheppard JP, Penaloza-Ramos CM, Jowett S, Ford GA, Lasserson D, Mant J, Mellor RM, Quinn T, Rothwell PM, et al. When has service provision for transient ischaemic attack improved enough? A discrete event simulation economic modelling study. *BMJ Open*. 2017;7:1–9.

15. Benavente L, Calleja S, Larrosa D, Vega J, Mauri G, Pascual J, Lahoz CH. Long term evolution of patients treated in a TIA unit. *Int. Arch. Med.* 2013;6:1–8.

© 2022 Shahjouei S et al. *JAMA Network Open.*

16. Birns J, Vilasuso M, Cohen DL. One-stop clinics are more effective than neurology clinics for TIA [2]. *Age Ageing* [Internet]. 2006;35:306–308. Available from: https://www.scopus.com/inward/record.uri?eid=2-s2.0-33646192887&doi=10.1093%2Fageing%2Fafj057&partnerID=40&md5=84d0ee05cced1607580b262dba886c44

17. Boden-Albala B, Goldmann E, Parikh NS, Carman H, Roberts ET, Lord AS, Torrico V, Appleton N, Birkemeier J, Parides M, et al. Efficacy of a Discharge Educational Strategy vs Standard Discharge Care on Reduction of Vascular Risk in Patients with Stroke and Transient Ischemic Attack: The DESERVE Randomized Clinical Trial. *JAMA Neurol.* 2019;76:20–27.

18. Bose P, Wilson A, Mistri A. Diagnosis and management of transient ischemic attacks in primary care: A systematic review. J. Prim. Health Care. 2017;9:114–130.

19. Bots ML, Wilk EC van der, Koudstaal PJ, Hofman A, Diederick E. Grobbee. Transient Neurological Attacks in the General Population. Prevalence, risk factors, and clinical relevance. *Stroke*. 1997;28:768–773.

20. Boulanger JM, Lindsay MP, Gubitz G, Smith EE, Stotts G, Foley N, Bhogal S, Boyle K, Braun L, Goddard T, et al. Canadian Stroke Best Practice Recommendations for Acute Stroke Management: Prehospital, Emergency Department, and Acute Inpatient Stroke Care, 6th Edition, Update 2018. *Int. J. Stroke*. 2018;13:949–984.

21. Bradley D, Cronin S, Kinsella JA, Tobin WO, Mahon C, O'Brien M, Lonergan R, Cooney MT, Kennelly S, Collins DR, et al. Frequent inaccuracies in ABCD2 scoring in non-stroke specialists' referrals to a daily Rapid Access Stroke Prevention service. *J. Neurol. Sci.* [Internet]. 2013;332:30–34. Available from: http://dx.doi.org/10.1016/j.jns.2013.05.030

22. Brainin M, McShane LM, Steiner M, Dachenhausen A, Seiser A. Silent brain infarcts and transient ischemic attacks: A three-year study of first-ever ischemic stroke patients:The klosterneuburg stroke data bank. *Stroke*. 1995;26:1348–1352.

23. Bravata DM, Myers LJ, Arling G, Miech EJ, Damush T, Sico JJ, Phipps MS, Zillich AJ, Yu Z, Reeves M, et al. Quality of care for veterans with transient ischemic attack and minor stroke. *JAMA Neurol.* 2018;75:419–427.

24. Bravata DM, Myers LJ, Reeves M, Cheng EM, Baye F, Ofner S, Miech EJ, Damush T, Sico JJ, Zillich A, et al. Processes of Care Associated with Risk of Mortality and Recurrent Stroke among Patients with Transient Ischemic Attack and Nonsevere Ischemic Stroke. *JAMA Netw. Open*. 2019;2:1–13.

25. Burn J, Dennis M, Bamford J, Sandercock P, Wade W, Warlow C. Erratum: Long-term risk of recurrent stroke after a first-ever stroke: The Oxfordshire Community Stroke Project. *Stroke*. 1994;25:333–337.

26. Johnston SC, Peter M Rothwell, Nguyen-Huynh MN, Giles MF, Elkins JS, Bernstein AL, Sidney S. Validation and refinement of scores to predict very early stroke risk after transient ishcaemic attack. *Lancet*. 2007;369:283–92.

27. Calvet D, Lamy C, Touzé E, Oppenheim C, Meder JF, Mas JL. Management and outcome of patients with transient ischemic attack admitted to a stroke unit. *Cerebrovasc. Dis.* 2007;24:80–85.

28. Calvet D, Touzé E, Oppenheim C, Turc G, Meder JF, Mas JL. DWI lesions and TIA etiology improve the prediction of stroke after TIA. *Stroke*. 2009;40:187–192.

29. Cameron AC, Dawson J, Quinn TJ, Walters MR, McInnes GT, Morrison D, Sloan W, Lees KR. Long-term outcome following attendance at a transient ischemic attack clinic. *Int. J. STROKE*. 2011;6:306–311.

30. Candelise L, Vigotti M, Fieschi C, Brambilla GL, Bono G, Conforti P, Zanche L De, Inzttari D, Mariani F, Prencipe M, et al. Italian multicenter study on reversible cerebral ischemic attacks: VI — Prognostic factors and follow-up results. *Stroke*. 1986;17:842–848.

31. Chandratheva A, Geraghty OC, Luengo-Fernandez R, Rothwell PM. ABCD2 score predicts severity rather than risk of early recurrent events after transient ischemic attack. *Stroke*. 2010;41:851–856.

32. Chang BP. Can I send this patient with stroke home? Strategies Managing Transient Ischemic Attack and

© 2022 Shahjouei S et al. *JAMA Network Open.*

Minor Stroke in the Emergency Department. *Physiol. Behav.* 2017;176:139–148.

33. Clissold B, Phan TG, Ly J, Singhal S, Srikanth V, Ma H. Current aspects of TIA management. *J. Clin. Neurosci.* [Internet]. 2020;72:20–25. Available from: https://doi.org/10.1016/j.jocn.2019.12.032

34. Cosker K, Samson S, Woimant F, Tuppin P. First hospitalization for transient ischemic attack in France : Characteristics , treatments and 3-year outcomes. *Rev. Neurol. (Paris).* [Internet]. 2015;172:152–159. Available from: http://dx.doi.org/10.1016/j.neurol.2015.06.008

35. Couillard P, Poppe AY, Coutts SB. Predicting recurrent stroke after minor stroke and transient ischemic attack. *Expert Rev. Cardiovasc. Ther.* 2009;7:1273–1281.

36. Coull AJ, Lovett JK, Rothwell PM, Oxford Vascular Study. Population based study of early risk of stroke after transient ischaemic attack or minor stroke: Implications for public education and organisation of services. *Br. Med. J.* 2004;328:326–328.

37. Cruz-Flores S. Acute Stroke and Transient Ischemic Attack in the Outpatient Clinic. *Med. Clin. North Am.* [Internet]. 2017;101:479–494. Available from: http://dx.doi.org/10.1016/j.mcna.2017.01.001

38. Dawson J, Lamb KE, Quinn TJ, Lees KR, Horvers M, Verrijth MJ, Walters MR. A recognition tool for transient ischaemic attack. *Qjm*. 2008;102:43–49.

39. Dennis MS, Bamford JM, Sandercock PAG, and C.P. Warlow. Incidence of transient ischemic attacks in Oxfordshire, England. *Stroke*. 1989;20:333–339.

40. Dennis M, Bamford J, Sandercock P, Warlow C. Prognosis of transient ischemic attacks in the Oxfordshire community stroke project. *Stroke*. 1990;21:848–853.

41. Dolmans LS, Hoes AW, Bartelink MLEL, Koenen NCT, Kappelle LJ, Rutten FH. Patient delay in TIA: a systematic review. *J. Neurol.* [Internet]. 2019;266:1051–1058. Available from: http://dx.doi.org/10.1007/s00415-018-8977-6

42. Dutta D. Diagnosis of TIA (DOT) score - design and validation of a new clinical diagnostic tool for transient ischaemic attack. *BMC Neurol.* [Internet]. 2016;16:1–7. Available from: http://dx.doi.org/10.1186/s12883-016-0535-1

43. Edlow JA. Managing Patients With Transient Ischemic Attack. *Ann. Emerg. Med.* [Internet]. 2018;71:409–415. Available from: https://doi.org/10.1016/j.annemergmed.2017.06.026

44. Eliasziw M, Streifler JY, Spence JD, Fox AJ, Hachinski VC, Barnett HJM. Prognosis for patients following a transient ischemic attack with and without a cerebral infarction on brain ct. *Neurology*. 1995;45:428–431.

45. Evans BA, Sicks JD, Whisnant JP. Factors Affecting Survival and Occurrence of Stroke in Patients With Transient Ischemic Attacks. *Mayo Clin. Proc.* 1994;69:416–421.

46. Falke P, Lindgärde F, Stavenow L. Prognostic indicators for mortality in transient ischemic attack and minor stroke. *Acta Neurol. Scand.* 1994;90:78–82.

47. Feigin VL, Shishkin S V., Tzirkin GM, Vinogradova TE, Tarasov A V., Vinogradov SP, Nikitin YP. A population-based study of transient ischemic attack incidence in Novosibirsk, Russia, 1987-1988 and 1996-1997. *Stroke*. 2000;31:9–13.

48. Ferro JM, Crespo M. Prognosis after transient ischemic attack and ischemic stroke in young adults. *Stroke*. 1994;25:1611–1616.

49. Fiorelli M, Argentino C, Sacchetti ML, Toni D, Sette G, Cavalletti C, Gori MC, Fieschi C, Alpérovitch A. Prediction of long-term outcome in the early hours following acute ischemic stroke. *Arch. Neurol.* 1995;52:250–255.

50. Floßmann E, Rothwell PM. Prognosis of vertebrobasilar transient ischaemic attack and minor stroke. *Brain*. 2003;126:1940–1954.

51. Fratiglioni L, Arfaioli C, Nencini P, Ginanneschi A, Iaquinta L, Marchi M, Inzitari D. Transient ischemic attacks in the community: Occurrence and clinical characteristics. A population survey in the area of

Florence, Italy. *Neuroepidemiology*. 1989;8:87–96.

52. Fratiglioni L, Inzitari D, Arfaioli C, Nencini P, Ginanneschi A, Pracucci G, Zuppiroli A, Italian G, Amaducci L. Risk factors for transient ischemic attacks in middle-age. A population-based case-control study. *Acta Neurol. Scand.* 1991;83:214–220.

53. Friday G, Alter M, Lai SM, Sobe E. Transient ischemic attack and risk of stroke recurrence: The Lehigh Valley Recurrent Stroke Study. *J. Stroke Cerebrovasc. Dis.* 1997;6:410–415.

54. Gennesseaux J, Giordano Orsini G, Lefour S, Bakchine S, Marion Q, Barbe C, Gennai S. Early Management of Transient Ischemic Attack in Emergency Departments in France. *J. Stroke Cerebrovasc. Dis.* 2019;29:104464.

55. Giles MF, Rothwell PM. Prediction and prevention of stroke after transient ischemic attack in the short and long term. *Expert Rev. Neurother.* 2006;6:381–395.

56. Giles MF, Rothwell PM. Risk of stroke early after transient ischaemic attack: a systematic review and meta-analysis. *Lancet. Neurol.* 2007;6:1063–72.

57. Giles MF, Rothwell PM. Substantial underestimation of the need for outpatient services for TIA and minor stroke. *Age Ageing*. 2007;36:676–680.

58. Gommans J, Barber PA, Fink J. Preventing strokes: The assessment and management of people with transient ischaemic attack. *N. Z. Med. J.* 2009;122:50–60.

59. Graham C, Bailey D, Hart S, Hutchison A, Sandercock P, Doubal F, Sudlow C, Farrall A, Wardlaw J, Dennis M, et al. Clinical diagnosis of TIA or minor stroke and prognosis in patients with neurological symptoms: A rapid access clinic cohort. *PLoS One*. 2019;14:e0210452.

60. Griffiths D, Sturm J, Heard R, Reyneke E, Whyte S, Clarke T, O'Brien W, Crimmins D. Can lower risk patients presenting with transient ischaemic attack be safely managed as outpatients? *J. Clin. Neurosci.* 2014;21:47–50.

61. Gulli G, Marquardt L, Rothwell PM, Markus HS. Stroke risk after posterior circulation stroke/transient ischemic attack and its relationship to site of vertebrobasilar stenosis: Pooled data analysis from prospective studies. *Stroke*. 2013;44:598–604.

62. Hankey GJ, Slattery JM, Warlow CP. The prognosis of hospital-referred transient ischaemic attacks. *J. Neurol. Neurosurg. Psychiatry*. 1991;54:793–802.

63. Hankey GJ, Slattery JM, Warlow CP. Transient ischaemic attacks: Which patients are at high (and low) risk of serious vascular events? *J. Neurol. Neurosurg. Psychiatry*. 1992;55:640–652.

64. Hayashi T, Kato Y, Nagoya H, Ohe Y, Deguchi I, Fukuoka T, Maruyama H, Horiuchi Y, Nagamine Y, Sano H, et al. Prediction of ischemic stroke in patients with tissue-defined transient ischemic attack. *J. Stroke Cerebrovasc. Dis.* 2014;23:1368–1373.

65. Hier DB, Foulkes MA, Swiontoniowski M, Sacco RL, Gorelick PB, Mohr JP, Price TR, Wolf PA. Stroke recurrence within 2 years after ischemic infarction. *Stroke*. 1991;22:155–161.

66. Holzer K, Feurer R, Sadikovic S, Esposito L, Bockelbrink A, Sander D, Hemmer B, Poppert H. Prognostic value of the ABCD2score beyond short-term follow-up after transient ischemic attack (TIA) - A cohort study. *BMC Neurol.* 2010;10:50.

67. Hörer S, Schulte-Altedorneburg G, Haberl RL. Management of patients with transient ischemic attack is safe in an outpatient clinic based on rapid diagnosis and risk stratification. *Cerebrovasc. Dis.* 2011;32:504–510.

68. Hoshino T, Mizuno S, Shimizu S, Uchiyama S. Clinical features and functional outcome of stroke after transient ischemic attack. *J. Stroke Cerebrovasc. Dis.* 2013;22:260–266.

69. Hoshino T, Nagao T, Mizuno S, Shimizu S, Uchiyama S. Transient neurological attack before vertebrobasilar stroke. *J. Neurol. Sci.* 2013;325:39–42.

70. Hosier GW, Phillips SJ, Doucette SP, Magee KD, Gubitz GJ. Transient ischemic attack: Management in

© 2022 Shahjouei S et al. *JAMA Network Open.*

the emergency department and impact of an outpatient neurovascular clinic. *Can. J. Emerg. Med.* 2016;18:331–339.

71. Howard G, Toole JF, Frye-Pierson J, Hinshelwood LC. Factors influencing the survival of 451 transient ischemic attack patients. *Stroke*. 1987;18:552–557.

72. Jeerakathil T, Shuaib A, Majumdar SR, Demchuk AM, Butcher KS, Watson TJ, Dean N, Gordon D, Edmond C, Coutts SB. The Alberta Stroke Prevention in TIAs and mild strokes (ASPIRE) intervention: Rationale and design for evaluating the implementation of a province-wide TIA Triaging system. *Int. J. Stroke*. 2014;9:135–143.

73. Johnson SE, Skre H. Transient cerebral ischemic attacks in the young and middle aged a population study. *Stroke*. 1986;17:662–666.

74. Johnston SC, Gress DR, Browner WS, Sidney S. Short-term prognosis after emergency department diagnosis of TIA. *J. Am. Med. Assoc.* 2000;284:2901–2906.

75. Joundi RA, Saposnik G. Organized Outpatient Care of Patients with Transient Ischemic Attack and Minor Stroke. *Semin. Neurol.* 2017;37:383–390.

76. Kandiyali R, Lasserson DS, Whiting P, Richards A, Mant J. Predictive values of referrals for transient ischaemic attack from first-contact health care: A systematic review. *Br. J. Gen. Pract.* 2017;67:e871–e880.

77. Kapral MK, Hall R, Fang J, Austin PC, Silver FL, Casaubon LK, Gladstone DJ, Stamplecoski M, Tu J V. Predictors of Hospitalization in Patients with Transient Ischemic Attack or Minor Ischemic Stroke. *Can. J. Neurol. Sci.* 2016;43:523–528.

78. Kernan WN, Viscoli CM, Brass LM, Makuch RW, Sarrel PM, Roberts RS, Gent M, Rothwell P, Sacco RL, Liu R-C, et al. The Stroke Prognosis Instrument II (SPI-II). *Stroke*. 2000;31:456–462.

79. Kimura K, Kazui S, Minematsu K, Yamaguchi T. Analysis of 16 , 922 Patients with Acute Ischemic Stroke and Transient Ischemic Attack in Japan. *Cerebrovasc Dis*. 2004;8565:47–56.

80. Kokubo Y. Epidemiology of transient ischemic attack. *Front. Neurol. Neurosci.* 2014;33:69–81.

81. Lai SM, Alter M, Friday G, Sobel E, Gil-Peralta A, McCoy RL, Levitt LP, Isack T. Transient ischemic attacks:Their Frequency in the lehigh valley. *Neuroepidemiology*. 1990;9:124–130.

82. Lavallée PC, Meseguer E, Abboud H, Cabrejo L, Olivot J, Simon O, Mazighi M, Nifl C, Niclot P, Lapergue B, et al. A transient ischaemic attack clinic with round-the-clock access (SOS-TIA): feasibility and effects. *Lancet Neurol*. 2007;6:953–960.

83. Lee W, Frayne J. Transient ischaemic attack clinic: An evaluation of diagnoses and clinical decision making. *J. Clin. Neurosci.* [Internet]. 2015;22:645–648. Available from: http://dx.doi.org/10.1016/j.jocn.2014.09.020

84. Lesenskyj AM, Maxwell CR, Veznedaroglu E, Liebman K, Hakma Z, Binning MJ. An Analysis of Transient Ischemic Attack Practices: Does Hospital Admission Improve Patient Outcomes? *J. Stroke Cerebrovasc. Dis.* [Internet]. 2016;25:2122–2125. Available from: http://dx.doi.org/10.1016/j.jstrokecerebrovasdis.2016.06.002

85. Levi CR, Lasserson D, Quain D, Valderas J, Dewey HM, Alan Barber P, Spratt N, Cadilhac DA, Feigin V, Zareie H, et al. The International comparison of Systems of care and patient outcomes In minor Stroke and Tia (InSIST) study: A community-based cohort study. *Int. J. Stroke*. 2019;14:186–190.

86. Loeb C, Priano A, Albano C. Clinical features and long-term follow-up of patients with reversible ischemic attacks (RIA). *Acta Neurol. Scand.* 1978;57:471–480.

87. Lovett JK, Dennis MS, Sandercock PAG, Bamford J, Warlow CP, Rothwell PM. Very early risk of stroke after a first transient ischemic attack. *Stroke*. 2003;34:e138-40.

88. Luengo-Fernandez R, Paul NLM, Gray AM, Pendlebury ST, Bull LM, Welch SJV, Cuthbertson FC, Rothwell PM. Population-based study of disability and institutionalization after transient ischemic attack and stroke: 10-year results of the oxford vascular study. *Stroke*. 2013;44:2854–2861.

© 2022 Shahjouei S et al. *JAMA Network Open.*

89. Madsen TE, Wira CR. The Future of Minor Stroke and Transient Ischemic Attack: The RAVEN Approach Is Promising but Not Ready for Prime Time. *Ann. Emerg. Med.* [Internet]. 2019;74:572–574. Available from: https://doi.org/10.1016/j.annemergmed.2019.08.458

90. Magin P, Lasserson D, Parsons M, Spratt N, Evans M, Russell M, Royan A, Goode S, McElduff P, Levi C. Referral and triage of patients with transient ischemic attacks to an acute access clinic: risk stratification in an Australian setting. *Int. J. STROKE*. 2013;8:81–89.

91. Martí-Vilalta JL, Lopez-Pousa S, Grau JM, Barraquer L. Transient ischemic attacks. Retrospective study of 150 cases of ischemic infarct in the territory of the middle cerebral artery. *Stroke*. 1979;10:259–262.

92. Mas JL, Zuber M. Recurrent cerebrovascular events in patients with patent foramen ovale, atrial septal aneurysm, or both and cryptogenic stroke or transient ischemic attack. *Am. Heart J.* 1995;130:1083–1088.

93. Mendelowitz DS, Kimmins S, Evans WE. Prognosis of patients with transient ischemic attacks and normal angiograms. *Arch. Surg.* 1981;116:1587–1591.

94. Merwick Á, Kelly PJ. Transient ischaemic attack clinics and management of transient ischaemic attacks. *Curr. Opin. Neurol.* 2011;24:50–58.

95. Mettinger KL, Söderström CE, Allander E. Epidemiology of acute cerebrovascular disease before the age of 55 in the stockholm county 1973-77: I incidence and mortality rates. *Stroke*. 1984;15:795–801.

96. Miller JFTRJKCRCCDFJHS. Transient Ischemic Attacks Due to Atherosclerosis. *Arch. Neurol.* 1975;32:5.

97. Mortel KF, Meyer JS. Prospective study of vascular events and cerebral perfusional changes following transient ischemic attacks. *Angiology*. 1996;47:215–224.

98. Muuronen A, Kaste M. Outcome of 314 patients with transient ischemic attacks. *Stroke*. 1982;13:24–31.

99. Norris JW. Outcome of Transient Ischaemic Attacks and Stroke. *Drugs*. 1991;42:10–15.

100. O'Brien EC, Zhao X, Fonarow GC, Schulte PJ, Dai D, Smith EE, Schwamm LH, Bhatt DL, Xian Y, Saver JL, et al. Quality of care and ischemic stroke risk after hospitalization for transient ischemic attack: Findings from get with the guidelines-stroke. *Circ. Cardiovasc. Qual. Outcomes*. 2015;8:S117–S124.

101. Ois A, Cuadrado-Godia E, Giralt-Steinhauer E, Jimenez-Conde J, Soriano-Tarraga C, Rodríguez-Campello A, Avellaneda C, Cascales D, Fernandez-Perez I, Roquer J. Long-term stroke recurrence after transient ischemic attack: Implications of etiology. *J. Stroke*. 2019;21:184–189.

102. Ong MEH, Chan YH, Lin WP, Chung WL. Validating the ABCD2 Score for predicting stroke risk after transient ischemic attack in the ED. *Am. J. Emerg. Med.* 2010;28:44–48.

103. Park HK, Kim BJ, Han MK, Park JM, Kang K, Lee SJ, Kim JG, Cha JK, Kim DH, Nah HW, et al. One-year outcomes after minor stroke or high-risk transient ischemic attack Korean multicenter stroke registry analysis. *Stroke*. 2017;48:2991–2998.

104. Pendlebury ST, Rothwell PM. Risk of recurrent stroke, other vascular events and dementia after transient ischaemic attack and stroke. *Cerebrovasc. Dis.* 2009;27:1–11.

105. Purroy F, Montserrat J, Begué R, Gil MI, Quílez A, Sanahuja J, Brieva L, Pardina M, Piñol-Ripoll G. Higher carotid intima media thickness predicts extracranial vascular events and not stroke recurrence among transient ischemic attack patients. *Int. J. Stroke*. 2012;7:125–132.

106. Purroy F, Jiménez Caballero PE, Gorospe A, Torres MJ, Alvarez-Sabin J, Santamarina E, Martínez-Sánchez P, Cánovas D, Freijo MJ, Egido JA, et al. Recurrent transient ischaemic attack and early risk of stroke: data from the PROMAPA study. *J. Neurol. Neurosurg. Psychiatry*. 2013;84:596–603.

107. Purroy F, Jiménez Caballero PE, Gorospe A, Torres MJ, Álvarez-Sabin J, Martínez-Sánchez P, Cánovas D, Freijo M, Egido JA, Ramírez-Moreno JM, et al. How predictors and patterns of stroke recurrence after a TIA differ during the first year of follow-up. *J. Neurol.* 2014;261:1614–1621.

108. Redgrave JNEE, Coutts SB, Schulz UG, Briley D, Rothwell PM. Systematic review of associations between the presence of acute ischemic lesions on diffusion-weighted imaging and clinical predictors of

early stroke risk after transient ischemic attack. *Stroke*. 2007;38:1482–1488.

109. Rhoads GG, Popper JS, Kagan A, Yano K. Incidence of transient cerebral ischemic attack in hawaii japanese men: The honolulu heart study. *Stroke*. 1980;11:21–36.

110. Rooij FG van, Dijk EJ van, Frank-Erik de Leeuw. 24/7 TIA-service. *Integr. Care Case Vol.* 2012;12.

111. Rothwell PM. Incidence, risk factors and prognosis of stroke and TIA: The need for high-quality, large-scale epidemiological studies and meta-analyses. *Cerebrovasc. Dis.* 2003;16:2–10.

112. Rothwell PM, Giles MF, Flossmann E, Lovelock CE, Redgrave JNEE, Warlow CP, Mehta Z. A simple score (ABCD) to identify individuals at high early risk of stroke after transient ischaemic attack. *Lancet*. 2005;366:29–36.

113. Rothwell PM, Warlow CP. Timing of TIAs preceding stroke: Time window for prevention is very short. *Neurology*. 2005;64:817–820.

114. Sales M, Quain D, Lasserson D, Levi C, Oldmeadow C, Jiwa M, Parsons M, Russell M, Clarey J, Magin P. Quality of referrals and guideline compliance for time to consultation at an acute neurovascular clinic. *J. Stroke Cerebrovasc. Dis.* [Internet]. 2015;24:874–880. Available from: http://dx.doi.org/10.1016/j.jstrokecerebrovasdis.2014.12.004

115. Sanossian N, Ovbiagele B. The risk of stroke within a week of minor stroke or transient ischemic attack. *Expert Opin. Pharmacother.* 2008;9:2069–2076.

116. Shah KH, Kleckner K, Edlow JA. Short-term Prognosis of Stroke Among Patients Diagnosed in the Emergency Department With a Transient Ischemic Attack. *Ann. Emerg. Med.* 2008;51:316–323.

117. Shah S, Cooper B. The epidemiology of stroke and transient ischaemia in Brisbane, Australia. *Ital. J. Neurol. Sci.* 1995;16:603–612.

118. Simonsen N, Christiansen HD, Heltberg A, Marquardsen J, Pedersen HE, Sørensen PS. Long-term prognosis after transient ischemic attacks. *Acta Neurol. Scand.* 1981;63:156–168.

119. Sobel E, Alter M, Davanipour Z, Friday G, McCoy R, Levitt LP, Isack T. Stroke in the lehigh valley: Combined risk factors for recurrent ischemic stroke. *Neurology*. 1989;39:669–672.

120. Stead LG, Bellolio MF, Suravaram S, Brown RD, Bhagra A, Gilmore RM, Boie ET, Decker WW. Evaluation of transient ischemic attack in an emergency department observation unit. *Neurocrit. Care*. 2009;10:204–8.

121. Stead LG, Suravaram S, Bellolio MF, Enduri S, Rabinstein A, Gilmore RM, Bhagra A, Manivannan V, Decker WW. An assessment of the incremental value of the ABCD2 score in the emergency department evaluation of transient ischemic attack. *Ann. Emerg. Med.* [Internet]. 2011;57:46–51. Available from: http://dx.doi.org/10.1016/j.annemergmed.2010.07.001

122. Streifler Y, Eliasziw M, Benavente OR, Harbison JW, Hachinski VC, Barnett HJM, Simard D. The Risk of Stroke in Patients With First-Ever Attacks and High-grade Carotid Stenosis. *Arch Neurol.* 1995;52:246–249.

123. Terént A, Andersson B. The Outcome of Patients with Transient Ischemic Attacks and Stroke Treated with Anticoagulants. *Acta Med. Scand.* 1980;208:359–365.

124. Terent A. Survival after stroke and transient ischemic attacks during the 1970s and 1980s. *Stroke*. 1989;20:1320–1326.

125. Thacker EL, Wiggins KL, Rice KM, Longstreth WT, Bis JC, Dublin S, Smith NL, Heckbert SR, Psaty BM. Short-term and long-term risk of incident ischemic stroke after transient ischemic attack. *Stroke*. 2010;41:239–243.

126. Tomari S, Magin P, Lasserson D, Quain D, Valderas JM, Dewey HM, Barber PA, Spratt NJ, Cadilhac DA, Feigin VL, et al. The Characteristics of Patients With Possible Transient Ischemic Attack and Minor Stroke in the Hunter and Manning Valley Regions, Australia (the INSIST Study). *Front. Neurol.* 2020;11:1–7.

© 2022 Shahjouei S et al. *JAMA Network Open.*

127. Toole JF, Yuson CP, Janeway R, Johnston F, Davis CL, Cordell AR, Howard G. Transient ischemic attacks: A prospective study of 225 patients. *Neurology*. 1978;28:746–753.

128. Torres Macho J, Peña Lillo G, Pérez Martínez D, González Mansilla A, Gámez Díez S, Mateo Alvarez S, García De Casasola G. Outcomes of atherothrombotic transient ischemic attack and minor stroke in an emergency department: Results of an outpatient management program. *Ann. Emerg. Med.* [Internet]. 2011;57:510–516. Available from: http://dx.doi.org/10.1016/j.annemergmed.2010.09.009

129. Tsuda Y, Kimura K, Yoneda S, Miyai M, Isaki Y, Abe H. Transient ischemic attacks: racial differences, treatment, and prognosis. *Neurol. Res.* 1983;5:103–117.

130. Ueda K, Kiyohara Y, Hasuo Y, Yanai T, Kawano H, Wada J, Kato I, Kajiwara E, Omae T, Fujishima M. Transient cerebral ischemic attacks in a japanese community, Hisayama, Japan. *Stroke*. 1987;18:844–848.

131. Valls J, Peiro-Chamarro M, Cambray S, Molina-Seguin J, Benabdelhak I, Purroy F. A current estimation of the early risk of stroke after transient ischemic attack: A systematic review and meta-analysis. *Cerebrovasc. Dis.* 2017;43:90–98.

132. Vandenbussche N, Demeestere J, Lemmens R. Evaluation and outcome of triage for patients with transient ischemic attack A two-year analysis of the TIA Clinic of the University Hospitals Leuven. 2017;2016–2017.

133. Vilanova MB, Mauri-Capdevila G, Sanahuja J, Quilez A, Piñol-Ripoll G, Begué R, Gil MI, Codina-Barios MC, Benabdelhak I, Purroy F. Prediction of myocardial infarction in patients with transient ischaemic attack. *Acta Neurol. Scand.* 2015;131:111–119.

134. Vrethem M, Johansson I. Prognosis in patients with transient ischaemic attacks and minor stroke associated with a normal angiogram. *Eur. Neurol.* 1990;30:203–206.

135. Walker J, Isherwood J, Eveson D, Naylor AR. Triaging TIA/minor stroke patients using the ABCD2 score does not predict those with significant carotid disease. *Eur. J. Vasc. Endovasc. Surg.* [Internet]. 2012;43:495–498. Available from: http://ovidsp.ovid.com/ovidweb.cgi?T=JS&PAGE=reference&D=med9&NEWS=N&AN=22377238

136. Warrior L, Prabhakaran S. Transient ischemic attack evaluation models: Hospitalization, same-day clinics, or rapid evaluation units. *Am. J. Ther.* 2011;18:45–50.

137. Weber R, Weimar C, Diener HC. Medical prevention of stroke and stroke recurrence in patients with TIA and minor stroke. *Expert Opin. Pharmacother.* [Internet]. 2009;10:1883–1894. Available from: https://www.scopus.com/inward/record.uri?eid=2-s2.0-68149099709&doi=10.1517%2F14656560903048934&partnerID=40&md5=e58b7c60e7fa982643f0757bebfd1505

138. Webster F, Saposnik G, Kapral MK, Fang J, O'Callaghan C, Hachinski V. Organized outpatient care: Stroke prevention clinic referrals are associated with reduced mortality after transient ischemic attack and ischemic stroke. *Stroke*. 2011;42:3176–3182.

139. Weimar C, Benemann J, Michalski D, Müller M, Luckner K, Katsarava Z, Weber R, Diener HC. Prediction of Recurrent Stroke and Vascular Death in Patients with Transient Ischemic Attack or Nondisabling Stroke: A Prospective Comparison of Validated Prognostic Scores. *Stroke*. 2010;41:487–493.

140. Weisberg LA. Clinical characteristics of transient is chemic attacks in black patients. *Neurology*. 1991;41:1410–1414.

141. Whisnant JP, Wiebers DO. Clinical Epidemiology of Transient Cerebral Ischemic Attacks (TIA) in the Anterioisr and Posterior Cerebral Circulation. In: Occlusive Cerebrovascular Disease: Diagnosis and Surgical Management. WB Saunders; 1987. p. 60–65.

142. Widjaja, E., S. N. Salam, P. D. Griffiths, C. Kamara, C. Doyle and GSV. Is the rapid assessment stroke clinic rapid enough in assessing transient ischaemic attack and minor stroke ? *J. Neurol. Neurosurg. Psychiatry*. 2004;76:141–142.

143. Wijk I Van, Kappelle LJ, Van Gijn J, Koudstaal PJ, Franke CL, Vermeulen M, Gorter JW, Algra A. Long-

© 2022 Shahjouei S et al. *JAMA Network Open.*

term survival and vascular event risk after transient ischaemic attack or minor ischaemic stroke: A cohort study. *Lancet*. 2005;365:2098–2104.

144. Wu CM, McLaughlin K, Lorenzetti DL, Hill MD, Manns BJ, Ghali WA. Early Risk of Stroke After Transient Ischemic Attack A Systematic Review and Meta-analysis. *Arch Intern Med.* 2007;167:2417–2422.

145. Zhong W, Geng N, Wang P, Li Z, Cao L. Prevalence, causes and risk factors of hospital readmissions after acute stroke and transient ischemic attack: a systematic review and meta-analysis. Neurol. Sci. 2016;37:1195–1202.

146. Ahmad O, Penglase RG, Chen MS, Harvey I, Hughes AR, Lueck CJ. A retrospective analysis of inpatient compared to outpatient care for the management of patients with transient ischaemic attack. *J. Clin. Neurosci.* 2013;20:988–992.

147. Al-Khaled M, Matthis C, Eggers J. The prognostic impact of the stroke unit care versus conventional care in treatment of patients with transient ischemic attack: a prospective population-based German study. *J. Vasc. Interv. Neurol.* 2013;5:22–226.

148. Al-Khaled M, Eggers J. Early Hospitalization of Patients with TIA: A Prospective, Population-based Study. *J. STROKE Cerebrovasc. Dis.* 2014;23:99–105.

149. Appelros P, Berglund MH, Ström JO. Long-term risk of stroke after transient ischemic attack. *Cerebrovasc. Dis.* 2017;43:25–30.

150. Arsava EM, Furie KL, Schwamm LH, Sorensen AG, Ay H. Prediction of early stroke risk in transient symptoms with infarction: Relevance to the new tissue-based definition. *Stroke*. 2011;42:2186–2190.

151. Ay H, Arsava EM, Johnston SC, Vangel M, Schwamm LH, Furie KL, Koroshetz WJ, Sorensen AG. Clinical- and imaging-based prediction of stroke risk after transient ischemic attack: The CIP model. *Stroke*. 2009;40:181–186.

152. Bonifati DM, Lorenzi A, Ermani M, Refatti F, Gremes E, Boninsegna C, Filipponi S, Orrico D. Carotid stenosis as predictor of stroke after transient ischemic attacks. *J. Neurol. Sci.* 2011;303:85–89.

153. Bos MJ, Van Rijn MJE, Witteman JCM, Hofman A, Koudstaal PJ, Breteler MMB. Incidence and prognosis of transient neurological attacks. *JAMA - J. Am. Med. Assoc.* [Internet]. 2007;298:2877–2885. Available from: https://www.scopus.com/inward/record.uri?eid=2-s2.0-37549038251&doi=10.1001%2Fjama.298.24.2877&partnerID=40&md5=a8ed06cbdc01ab2713c107c1f62533e6

154. Cancelli I, Janes F, Gigli GL, Perelli A, Zanchettin B, Canal G, D'Anna L, Russo V, Barbone F, Valente M. Incidence of transient ischemic attack and early stroke risk: Validation of the ABCD2 score in an Italian population-based study. *Stroke*. 2011;42:2751–2757.

155. Chang BP, Rostanski S, Willey J, Miller EC, Shapiro S, Mehendale R, Kummer B, Navi BB, Elkind MS V. Safety and Feasibility of a Rapid Outpatient Management Strategy for Transient Ischemic Attack and Minor Stroke: The Rapid Access Vascular Evaluation-Neurology (RAVEN) Approach. *Ann. Emerg. Med.* [Internet]. 2019;74:562–571. Available from: https://ezproxy.uthsc.edu/login?url=http://search.ebscohost.com/login.aspx?direct=true&db=ccm&AN=138728145&site=ehost-live

156. Cheong E, Toner P, Dowie G, Jannes J, Kleinig T. Evaluation of a CTA-Triage Based Transient Ischemic Attack Service: A Retrospective Single Center Cohort Study. *J. Stroke Cerebrovasc. Dis.* [Internet]. 2018;27:3436–3442. Available from: https://doi.org/10.1016/j.jstrokecerebrovasdis.2018.08.006

157. Correia M, Fonseca AC, Canhão P. Short-term outcome of patients with possible transient ischemic attacks: A prospective study. *BMC Neurol.* 2015;15:78.

158. Coutts SB, Hill MD, Campos CR, Choi YB, Subramaniam S, Kosior JC, Demchuk AM. Recurrent events in transient ischemic attack and minor stroke: What events are happening and to which patients? *Stroke*. 2008;39:2461–2466.

159. Cucchiara BL, Messe SR, Sansing L, MacKenzie L, Taylor RA, Pacelli J, Shah Q, Kasner SE. Lipoprotein-Associated Phospholipase A(2) and C-Reactive Protein for Risk-Stratification of Patients With

© 2022 Shahjouei S et al. *JAMA Network Open.*

TIA. *STROKE*. 2009;40:2332–2336.

160. Dahlquist RT, Young JM, Reyner K, Farzad A, Moleno RB, Gandham G, Ho AF, Wang H. Initiation of the ABCD3-I algorithm for expediated evaluation of transient ischemic attack patients in an emergency department. *Am. J. Emerg. Med.* [Internet]. 2019;Available from: https://www.scopus.com/inward/record.uri?eid=2-s2.0-85067471067&doi=10.1016%2Fj.ajem.2019.06.018&partnerID=40&md5=358afef372e206b2bbc85f36f2ca9a62

161. De Marchis GM, Weck A, Audebert H, Benik S, Foerch C, Buhl D, Schuetz P, Jung S, Seiler M, Morgenthaler NG, et al. Copeptin for the Prediction of Recurrent Cerebrovascular Events After Transient Ischemic Attack Results From the CoRisk Study. *STROKE*. 2014;45:2918+.

162. Delgado P, Chacón P, Penalba A, Pelegri D, García-Berrocoso T, Giralt D, Santamarina E, Ribó M, Maisterra O, Alvarez-Sabín J, et al. Lipoprotein-associated phospholipase A 2 activity is associated with large-artery atherosclerotic etiology and recurrent stroke in TIA patients. *Cerebrovasc. Dis.* 2012;33:150–158.

163. Eliasziw M, Kennedy J, Hill MD, Buchan AM, Barnett HJM. Early risk of stroke after a transient ischemic attack in patients with internal carotid artery disease. *CMAJ*. 2004;170:1105–1109.

164. Engelter ST, Amort M, Jax F, Weisskopf F, Katan M, Burow A, Bonati LH, Hatz F, Wetzel SG, Fluri F, et al. Optimizing the risk estimation after a transient ischaemic attack - the ABCDE circle plus score. *Eur. J. Neurol.* 2012;19:55–61.

165. Felgueiras R, Magalhães R, Silva MR, Silva MC, Correia M. Transient ischemic attack: Incidence and early risk of stroke in northern Portugal from 1998–2000 to 2009–2011. *Int. J. Stroke*. 2019;0:1–11.

166. Fujinami J, Uehara T, Kimura K, Okada Y, Hasegawa Y, Tanahashi N, Suzuki A, Takagi S, Nakagawara J, Arii K, et al. Incidence and predictors of ischemic stroke events during hospitalization in patients with transient ischemic attack. *Cerebrovasc. Dis.* 2014;37:330–335.

167. Geil K, González-Concepción JJ, Jiménez-Velázquez IZ, Medina B, Velazco X. Management and outcome of transient ischemic attacks in Ponce, Puerto Rico. *Bol. Asoc. Med. P. R.* 2008;100:11–14.

168. Ghia D, Thomas P, Cordato D, Epstein D, Beran RG, Cappelen-Smith C, Griffith N, Hanna I, Mcdougall A, Hodgkinson SJ, et al. Low positive predictive value of the ABCD2 score in emergency department transient ischaemic attack diagnoses: The South Western Sydney Transient Ischaemic Attack Study. *Intern. Med. J.* 2012;42:913–918.

169. DJ G, MK K, Fang J, Laupacis A, JV T, Gladstone DJ, Kapral MK, Fang J, Laupacis A, Tu J V. Management and outcomes of transient ischemic attacks in Ontario. *C. Can. Med. Assoc. J.* [Internet]. 2004;170:1099–1104. Available from: https://ezproxy.uthsc.edu/login?url=http://search.ebscohost.com/login.aspx?direct=true&db=ccm&AN=106756735&site=ehost-live

170. Gon Y, Sakaguchi M, Okazaki S, Mochizuki H, Kitagawa K. Prevalence of positive diffusion-weighted imaging findings and ischemic stroke recurrence in transient ischemic attack. *J. Stroke Cerebrovasc. Dis.* [Internet]. 2015;24:1000–1007. Available from: http://dx.doi.org/10.1016/j.jstrokecerebrovasdis.2014.12.023

171. Guarino M, Rondelli F, Favaretto E, Stracciari A, Filippini M, Rinaldi R, Zele I, Sartori M, Faggioli G, Mondini S, et al. Short- and Long-Term Stroke Risk after Urgent Management of Transient Ischaemic Attack: The Bologna TIA Clinical Pathway. *Eur. Neurol.* 2015;74:1–7.

172. Harrison JK, Sloan B, Dawson J, Lees KR, Morrison DS. The ABCD and ABCD2 as predictors of stroke in transient ischemic attack clinic outpatients: A retrospective cohort study over 14 years. *Q J Med*. 2010;103:679–685.

173. Hill MD, Yiannakoulias N, Jeerakathil T, Tu J V, Svenson LW, Schopflocher DP. The high risk of stroke immediately after transient ischemic attack: A population-based study. *Neurology*. 2004;62:2015–2020.

174. Ildstad F, Ellekjaer H, Wethal T, Lydersen S, Sund JK, Fjaertoft H, Schuler S, Horn JW, Brathen G, Midtsaether A-G, et al. Stroke risk after transient ischemic attack in a Norwegian prospective cohort. *BMC*

© 2022 Shahjouei S et al. *JAMA Network Open.*

Neurol. 2019;19:2.

175. Johnston SC, Rothwell PM, Nguyen-Huynh MN, Giles MF, Elkins JS, Bernstein AL, Sidney S. Validation and refinement of scores to predict very early stroke risk after transient ischaemic attack. *Lancet*. 2007;369:283–292.

176. Jove M, Mauri-Capdevila G, Suarez I, Cambray S, Sanahuja J, Quilez A, Farre J, Benabdelhak I, Pamplona R, Portero-Otin M, et al. Metabolomics predicts stroke recurrence after transient ischemic attack. *Neurology*. 2015;84:36–45.

177. Kiyohara T, Kamouchi M, Kumai Y, Ninomiya T, Hata J, Yoshimura S, Ago T, Okada Y, Kitazono T, Investigators FSR. ABCD3 and ABCD3-I Scores Are Superior to ABCD2 Score in the Prediction of Short- and Long-Term Risks of Stroke After Transient Ischemic Attack. *STROKE*. 2014;45:418–425.

178. Kleindorfer D, Panagos P, Pancioli A, Khoury J, Kissela B, Woo D, Schneider A, Alwell K, Jauch E, Miller R, et al. Incidence and short-term prognosis of transient ischemic attack in a population-based study. *Stroke*. 2005;36:720–3.

179. Lichtman JH, Jones SB, Watanabe E, Allen NB, Wang Y, Howard VJ, Goldstein LB. Elderly Women Have Lower Rates of Stroke, Cardiovascular Events, and Mortality After Hospitalization for Transient Ischemic Attack. *Stroke* [Internet]. 2009;40:2116–2122. Available from: https://www.scopus.com/inward/record.uri?eid=2-s2.0-66849095968&doi=10.1161%2FSTROKEAHA.108.543009&partnerID=40&md5=e63fef8867561ab40210751a58ba10cf

180. Lim J-S, Hong K-S, Kim G-M, Bang OY, Bae H-J, Kwon H-M, Park J-M, Lee S-H, Rha J-H, Koo J, et al. Cerebral microbleeds and early recurrent stroke after transient ischemic attack: results from the Korean Transient Ischemic Attack Expression Registry. *JAMA Neurol.* 2015;72:301–8.

181. Lisabeth LD, Ireland JK, Risser JMH, Brown DL, Smith MA, Garcia NM, Morgenstern LB. Stroke risk after transient ischemic attack in a population-based setting. *Stroke*. 2004;35:1842–6.

182. Rothrock J, Majidi S, Burger K, Leon-Guererro C. Inpatient versus Outpatient Management of TIA or Minor Stroke: Clinical Outcome. *Neurology*. 2016;86.

183. Montassier E, Lim T-X, Goffinet N, Guillon B, Segard J, Martinage A, Potel G, Le Conte P. Results of an outpatient transient ischemic attack evaluation: a 90-day follow-up study. *J. Emerg. Med.* [Internet]. 2013;44:970–975. Available from: https://ezproxy.uthsc.edu/login?url=http://search.ebscohost.com/login.aspx?direct=true&db=ccm&AN=104283594&site=ehost-live

184. Nahab F, Leach G, Kingston C, Mir O, Abramson J, Hilton S, Keadey M, Gartland B, Ross M. Impact of an emergency department observation unit transient ischemic attack protocol on length of stay and cost. *J. Stroke Cerebrovasc. Dis.* 2012;21:673–678.

185. Nguyen H, Kerr D, Kelly AM. Comparison of prognostic performance of scores to predict risk of stroke in ED patients with transient ischaemic attack. *Eur. J. Emerg. Med.* 2010;17:346–348.

186. Ohara T, Uehara T, Toyoda K, Suzuki R, Sato S, Nagatsuka K, Minematsu K. Early stroke risk after transient ischemic attack in patients without large-artery disease or atrial fibrillation. *J. Stroke Cerebrovasc. Dis.* 2015;24:1656–61.

187. Ohara T, Uehara T, Sato S, Hayakawa M, Kimura K, Okada Y, Hasegawa Y, Tanahashi N, Suzuki A, Nakagawara J, et al. Small vessel occlusion is a high-risk etiology for early recurrent stroke after transient ischemic attack. *Int. J. STROKE*. 2019;14:871–877.

188. Olivot JM, Wolford C, Castle J, Mlynash M, Schwartz NE, Lansberg MG, Kemp S, Albers GW. TWO ACES: Transient ischemic attack work-up as outpatient assessment of clinical evaluation and safety. *Stroke*. 2011;42:1839–1843.

189. Ottaviani M, Vanni S, Moroni F, Peiman N, Boddi M, Grifoni S. Urgent carotid duplex and head computed tomography versus ABCD2 score for risk stratification of patients with transient ischemic attack. *Eur. J. Emerg. Med.* 2016;23:19–23.

© 2022 Shahjouei S et al. *JAMA Network Open.*

190. Ovbiagele B, Cruz-Flores S, Lynn MJ, Chimowitz MI. Early stroke risk after transient ischemic attack among individuals with symptomatic intracranial artery stenosis. *Arch. Neurol.* 2008;65:733–737.

191. Palomeras Soler E, Fossas Felip P, Cano Orgaz AT, Sanz Cartagena P, Casado Ruiz V, Muriana Batista D. Evaluación rápida del ataque isquémico transitorio en un hospital sin guardias de neurología. *Neurología*. 2015;30:325–330.

192. Perry JJ, Sharma M, Sivilotti MLA, Sutherland J, Worster A, Émond M, Stotts G, Jin AY, Oczkowski WJ, Sahlas DJ, et al. A prospective cohort study of patients with transient ischemic attack to identify high-risk clinical characteristics. *Stroke*. 2014;45:92–100.

193. Purroy F, Jimenez Caballero PE, Gorospe A, Torres MJ, Alvarez-Sabin J, Martinez-Sanchez P, Canovas D, Freijo M, Egido JA, Ramirez-Moreno JM, et al. How predictors and patterns of stroke recurrence after a TIA differ during the first year of follow-up. *J. Neurol.* 2014;261:1614–1621.

194. Raposo N, Albucher JF, Rousseau V, Acket B, Chollet F, Olivot JM. ED Referral Dramatically Reduces Delays of Initial Evaluation in a French TIA Clinic. *Front. Neurol.* 2018;9.

195. Raser JM, Cucchiara BL. Modifications of the ABCD(2) Score Do Not Improve the Risk Stratification of Transient Ischemic Attack Patients. *J. STROKE \& Cerebrovasc. Dis.* [Internet]. 2012;21:467–470. Available from: http://ovidsp.ovid.com/ovidweb.cgi?T=JS&PAGE=reference&D=med9&NEWS=N&AN=21388829

196. Ricci S, Celani MG, Rosa F La, Vitali R, Duca E, Ferraguzzi R, Paolotti M, Seppoloni D, Caputo N, Chiurulla C, et al. A community-based study of incidence, risk factors and outcome of transient ischaemic attacks in Umbria, Italy: the SEPIVAC study. *J. Neurol.* 1991;238:87–90.

197. Ross MA, Compton S, Medado P, Fitzgerald M, Kilanowski P, O'Neil BJ. An Emergency Department Diagnostic Protocol for Patients With Transient Ischemic Attack: A Randomized Controlled Trial. *Ann. Emerg. Med.* 2007;50:109–119.

198. Rothwell PM, Giles MF, Chandratheva A, Marquardt L, Geraghty O, Redgrave JN, Lovelock CE, Binney LE, Bull LM, Cuthbertson FC, et al. Effect of urgent treatment of transient ischaemic attack and minor stroke on early recurrent stroke (EXPRESS study): a prospective population-based sequential comparison. *Lancet*. 2007;370:1432–1442.

199. Sanders LM, Srikanth VK, Jolley DJ, Sundararajan V, Psihogios H, Wong K, Ramsay D, Phan TG. Monash transient ischemic attack triaging treatment: Safety of a transient ischemic attack mechanism-based outpatient model of care. *Stroke*. 2012;43:2936–2941.

200. Sciolla R, Melis F, Grp S. Rapid identification of high-risk transient ischemic attacks - Prospective validation of the ABCD score. *STROKE*. 2008;39:297–302.

201. Selvarajah JR, Smith CJ, Hulme S, Georgiou RF, Vail A, Tyrrell PJ. Prognosis in patients with transient ischaemic attack (TIA) and minor stroke attending TIA services in the North West of England: The NORTHSTAR Study. *J. Neurol. Neurosurg. Psychiatry*. 2008;79:38–43.

202. Song B, Fang H, Zhao L, Gao Y, Tan S, Lu J, Sun S, Chandra A, Wang R, Xu Y. Validation of the ABCD3-I score to predict stroke risk after transient ischemic attack. *Stroke* [Internet]. 2013;44:1244–1248. Available from: http://ovidsp.ovid.com/ovidweb.cgi?T=JS&PAGE=reference&D=med10&NEWS=N&AN=23532014

203. Stead LG, Suravaram S, Bellolio MF, Enduri S, Rabinstein A, Gilmore RM, Bhagra A, Manivannan V, Decker WW. An Assessment of the Incremental Value of the ABCD2 Score in the Emergency Department Evaluation of Transient Ischemic Attack. *Ann. Emerg. Med.* [Internet]. 2011;57:46–51. Available from: https://ezproxy.uthsc.edu/login?url=http://search.ebscohost.com/login.aspx?direct=true&db=ccm&AN=57074063&site=ehost-live

204. Sundararajan V, Thrift AG, Phan TG, Choi PM, Clissold B, Srikanth VK. Trends over time in the risk of stroke after an incident transient ischemic attack. *Stroke*. 2014;45:3214–8.

205. Tsivgoulis G, Stamboulis E, Sharma VK, Heliopoulos I, Voumvourakis K, Teoh HL, Patousi A, Andrikopoulou A, Lim EL, Stilou L, et al. Multicenter external validation of the ABCD(2) score in triaging

© 2022 Shahjouei S et al. *JAMA Network Open.*

TIA patients. *Neurology*. 2010;74:1351–1357.

206. Vigen T, Thommessen B, Rønning OM. Stroke risk is low after urgently treated transient ischemic attack. *J. Stroke Cerebrovasc. Dis.* 2018;27:291–295.

207. Vora N, Tung CE, Mlynash M, Garcia M, Kemp S, Kleinman J, Zaharchuk G, Albers G, Olivot J-M. TIA Triage in Emergency Department Using Acute MRI (TIA-TEAM): A Feasibility and Safety Study. *Int. J. Stroke*. 2015;10:343–347.

208. Wasserman J, Perry J, Dowlatshahi D, Stotts G, Stiell I, Sutherland J, Symington C, Sharma M. Stratified, Urgent Care for Transient Ischemic Attack Results in Low Stroke Rates. *STROKE*. 2010;41:2601–2605.

209. Weimar C, Benemann J, Huber R, Mieck T, Kaendler S, Grieshammer S, Katsarava Z, Diener HC. Long-term mortality and risk of stroke after transient ischemic attack : AAA hospital-based cohort study. *J. Neurol.* 2009;256:639–644.

210. Weitzel-Mudersbach P V, Johnsen SP, Andersen G, v. Weitzel-Mudersbach P, Johnsen SP, Andersen G. Low risk of vascular events following urgent treatment of transient ischaemic attack: The Aarhus TIA study. *Eur. J. Neurol.* 2011;18:1285–1290.

211. Wu CM, Manns BJ, Hill MD, Ghali WA, Donaldson C, Buchan AM. Rapid evaluation after high-risk TIA is associated with lower stroke risk. *Can. J. Neurol. Sci.* 2009;36:450–455.

© 2022 Shahjouei S et al. *JAMA Network Open.*
